# Polar Lipids of Commercial *Ulva* spp. of Different Origins: Profiling and Relevance for Seaweed Valorization

**DOI:** 10.3390/foods10050914

**Published:** 2021-04-21

**Authors:** Ana S. P. Moreira, Elisabete da Costa, Tânia Melo, Diana Lopes, Adriana C. S. Pais, Sónia A. O. Santos, Bárbara Pitarma, Madalena Mendes, Maria H. Abreu, Pi Nyvall Collén, Pedro Domingues, M. Rosário Domingues

**Affiliations:** 1 Department of Chemistry, Santiago University Campus, CICECO-Aveiro Institute of Materials, University of Aveiro, 3810-193 Aveiro, Portugal; a.c.p.s@ua.pt (A.C.S.P.); santos.sonia@ua.pt (S.A.O.S.); 2 Department of Chemistry, LAQV-REQUIMTE, Santiago University Campus, University of Aveiro, 3810-193 Aveiro, Portugal; elisabetecosta@ua.pt (E.d.C.); taniamelo@ua.pt (T.M.); dianasalzedaslopes@ua.pt (D.L.); p.domingues@ua.pt (P.D.); mrd@ua.pt (M.R.D.); 3 CESAM-Centre for Environmental and Marine Studies, Department of Chemistry, Santiago University Campus, University of Aveiro, 3810-193 Aveiro, Portugal; 4 ALGAplus-Produção e Comercialização de Algas e seus Derivados, 3830-196 Ílhavo, Portugal; barbara.pitarma@algaplus.pt (B.P.); madalena.mendes@algaplus.pt (M.M.); helena.abreu@algaplus.pt (M.H.A.); 5 Green Colab—Associação Oceano Verde, Campus de Gambelas, University of Algarve, 8005-139 Faro, Portugal; 6 Amadeite SAS, Pôle Biotechnologique du Haut du Bois, 56580 Bréhan, France; PNyvallCollen@olmix.com

**Keywords:** algae, sea lettuce, glycolipids, phospholipids, betaine lipids, IMTA, wild

## Abstract

Macroalgae of the genus *Ulva* have long been used as human food. Local environmental conditions, among other factors, can have an impact on their nutrient and phytochemical composition, as well as on the value of the seaweed for food and non-food applications. This study is the first to initiate a comparison between commercial *Ulva* spp. from different European origins, France (FR, wild-harvested *Ulva* spp.), and Portugal (PT, farm-raised *Ulva rigida*), in terms of proximate composition, esterified fatty acids (FA), and polar lipids. The ash content was higher in PT samples, while FR samples had higher levels of proteins, lipids, and carbohydrates and other compounds. The profile of esterified FA, as well as FA-containing polar lipids at the class and species levels were also significantly different. The FR samples showed about three-fold higher amount of *n*-3 polyunsaturated FA, while PT samples showed two-fold higher content of monounsaturated FA. Quantification of glycolipids and phospholipids revealed, respectively, two-fold and three-fold higher levels in PT samples. Despite the differences found, the polar lipids identified in both batches included some lipid species with recognized bioactivity, valuing *Ulva* biomass with functional properties, increasing their added value, and promoting new applications, namely in nutraceutical and food markets.

## 1. Introduction

Given the negative environmental impact of current food systems, there is an urgent need to promote healthy and sustainable diets with low environmental impacts [1]. In this context, along with the fast-growing world population driving up global food demand, marine macroalgae (commonly known as seaweed) are of growing interest, as natural and sustainable sources of essential nutrients and health-promoting compounds [2,3].

Macroalgae contain a wide range of valuable compounds, such as polysaccharides, lipids, proteins, phenolic compounds, and pigments [2,4]. The composition of these compounds is influenced by natural fluctuations in environmental parameters (e.g., temperature, light, salinity, and availability of nutrients), as well as by biotic interactions [4,5,6,7]. Lipids, despite their low content (less than 5% of the dry matter of several macroalgae) [3,8], are considered a valuable source of polyunsaturated fatty acids (PUFA), namely omega-3 (*n*-3) PUFA, known for their beneficial health impacts [9,10]. Most PUFA are generally esterified into different classes of polar lipids, including glycolipids, betaine lipids, and phospholipids [5,7]. Glycolipids are the most recognized for their bioactive properties and potential health benefits, namely as antioxidant, anti-inflammatory, antimicrobial, and antitumoral compounds [11,12,13,14,15,16,17]. Despite the increasing research on these compounds, polar lipids of macroalgae remain a largely unknown reservoir of molecular diversity [17]. This diversity needs to be explored, promoting the valorization of these marine resources and the development new applications.

Due to their composition in lipids and other bioactive compounds, macroalgae are excellent candidates for use as food and raw material for multiple applications, contributing to the development of high-value markets such as functional foods, cosmeceuticals, nutraceuticals, and pharmaceuticals [2,18,19,20,21]. They also represent potential agents to combat non-communicable diseases such as type 2 diabetes, obesity, and cardiovascular disease [3,22]. Indeed, seaweed production has increased significantly in response to its wider use in the food and non-food commercial industries [23].

Among commercialized macroalgae, *Ulva* species (commonly known as sea lettuce) have long been used as food. Commercially available *Ulva* species can be collected from wild populations, but they are increasingly cultivated in outdoor facilities [24,25,26]. In particular, *Ulva* species are successfully produced year-round in land-based integrated multi-trophic aquaculture (IMTA) systems [25], which provide a sustainable and environmentally friendly alternative to reduce the potential impact and dependence from wild populations. Cultivation of seaweed on land-based systems also allows control of growth conditions, which is considered advantageous for producing high-value seaweed biomass with consistent properties and chemical composition [25,27]. It should be noted that commercial *Ulva* species are generally labeled as sea lettuce or *Ulva* spp. (with no scientific name) because the taxonomy for this genus is challenging [24], and it is economically unviable to genotype each batch on the market, especially for batches collected in the wild.

Whether in wild populations or in cultivation, local environmental conditions, among other factors, can impact the composition of *Ulva* spp. and hence the uses of seaweed for applications in food and non-food industries. Previous studies have shown changes in the lipids of *Ulva* spp. when exposed to different environmental conditions, whether in different seasons [28,29,30,31,32] or geographic locations [33]. In most of these studies, only changes in lipid content and fatty acid composition were evaluated. Although increasingly recognized as a source of variability, the impact of natural fluctuations in the environment on the polar lipidome of *Ulva* spp. at the molecular level has not been actively investigated, with only two published studies [29,33]. Further studies are needed to understand the range of natural variability of polar lipid species, targeting the premium food market and high-value industries. In-depth knowledge of variations in the lipidome of *Ulva* spp. from different geographical origins are particularly important for the valorization of specific production sites, creating new marketing and economic development opportunities. Also, it can be useful for the authentication and certification of the geographical origin of *Ulva* species [33]. On other hand, the natural variability related to species-specific differences, life-cycle stage, and biotic interactions [34,35,36], as well as the impact of cultivation and processing protocols [37,38], cannot be excluded when considering polar lipids from commercial *Ulva* spp. and their promising applications.

In this study, commercial *Ulva* spp. from two different European sites, Portugal (PT, farm-raised *Ulva rigida*) and France (FR, wild-harvested *Ulva* spp.), were harvested in May and compared in terms of proximate composition and detailed polar lipid composition. The extracts enriched in polar lipids were analyzed using high-resolution hydrophilic interaction liquid chromatography-mass spectrometry and tandem mass spectrometry (HILIC–MS and MS/MS) for profiling polar lipids, either at the level of lipid classes or lipid species. Additionally, gas chromatography-mass spectrometry (GC–MS) was used for profiling esterified FA. The evaluation of the variations between PT and FR samples was performed using multivariate and univariate statistical analysis.

## 2. Materials and Methods

### 2.1. Samples

Dried samples of *Ulva* spp. were provided by ALGAplus (Ílhavo, Portugal) and Olmix (Bréhan, France), both harvested in May 2017. ALGAplus produces *Ulva rigida* in an open land-based integrated multi-trophic aquaculture (IMTA) system located at Ria de Aveiro coastal lagoon (Portugal, 40°36′43″ N 8°40′43″ W) (PT samples). Olmix harvests wild *Ulva* spp. in the north Brittany coast near Plestin les Grèves (France, 48°40′49.9″ N 3°35′40.1″ W) (FR samples). Each macroalgae batch was washed and dried according to the respective internal procedures.

### 2.2. Proximate Composition Analysis

The moisture content of freeze-dried and milled biomass was determined by drying samples (250 mg × 5 replicates) at 105 °C for 15 h, which were then placed in a muffle furnace at 575 °C for a duration of 6 h for ash determination [39]. Elemental analysis (C, H, N, and S) (2 mg × 5 replicates) was performed on a Leco TruspecMicro CHNS 630-200-200 elemental analyzer using a combustion furnace temperature of 1075 °C and afterburner temperature of 850 °C. Two nitrogen-protein conversion factors, 6.25 and 5.0, were used to calculate the protein content [40]. Lipid content was determined by gravimetry of polar lipid extracts (Section 2.3). The content of carbohydrates and other compounds was estimated by difference, subtracting the percentage of ash, proteins (N × 6.25), and lipids from 100%. All contents were expressed as a percentage of the dry weight (%DW), excluding moisture (% of freeze-dried sample weight).

### 2.3. Extraction of Polar Lipids

Extracts containing polar lipids were obtained from *Ulva* spp. samples using the Bligh and Dyer method [41], with slight modifications [29,39]. Dried samples (250 mg × 5 replicates) were homogenized by adding methanol (2.5 mL) and chloroform (1.25 mL), vortexing for 2 min, and applying sonication for 1 min, followed by incubation in ice with shaking for 2.5 h. The samples were then centrifuged for 10 min at 2000 rpm. After the recovery of the organic phase, the biomass residue was re-extracted with methanol (2 mL) and chloroform (1 mL). The combined organic phase was washed by adding Milli-Q water (2.3 mL), followed by centrifugation as above. The aqueous phase was washed with chloroform (2 mL), whereas the biomass residue was re-extracted again with methanol (2 mL) and chloroform (1 mL). The final organic phase (denominated as polar lipid extract) was dried to dryness, resuspended in chloroform, transferred to amber vials (previously weighed), dried again under nitrogen, weighed, and stored at −20 °C before analysis.

### 2.4. GC–MS Analysis of Esterified Fatty Acids

To obtain fatty acid methyl ester (FAME) derivatives by base-catalyzed transmethylation [42], dried lipid extracts (30 μg) were mixed with 1 mL of an internal standard solution (1.0 μg mL^−1^ of methyl nonadecanoate in n-hexane) and 200 μL of a KOH solution (2 mol L^−1^ in methanol). After vortexing for 2 min, 2 mL of a NaCl solution (10 mg mL^−1^ in water) were added and the sample was centrifuged for 5 min at 2000 rpm. A volume of 600 µL of the organic (upper) phase containing FAME derivatives was recovered and dried to dryness under nitrogen. FAME were then dissolved in 60 μL of n-hexane and 2 μL of this solution were used for gas chromatography-mass spectrometry (GC–MS) analysis (6890N Network GC system and 5973 Network Mass Selective Detector; Agilent Technologies, Santa Clara, CA, USA). The GC system was equipped with a DB-FFAP (J&W Scientific, Folsom, CA, USA) capillary column (30 m × 0.32 mm internal diameter, 0.25 µm film thickness) and used helium (1.4 mL min^−1^) as carrier gas, with injector temperature of 220 °C, detector temperature of 280 °C, and oven temperature of 80 °C (3 min) → 25 °C min^−1^ → 160 °C → 2 °C min^−1^ → 210 °C → 30 °C min^−1^ → 250 °C (10 min). The MS system was operated with electron impact ionization at 70 eV in full scan mode, scanning *m*/*z* 50–550 in a 1 s cycle. The FAME derivatives were identified by comparing their retention times and mass spectra with those of a commercial mixture of FAME standards (Supelco 37 Component FAME Mix, Sigma-Aldrich, Darmstadt, Germany) and by comparing with the available MS spectra in the Wiley 275 library and The Lipid Web library [43], the latter containing all FA found in the *Ulva* samples. For FA quantification, calibration curves were obtained under the same instrumental conditions from different dilutions of the FAME mixture (dilution factor of 10 to 400), all containing the same amount of internal standard (3.7 μg of methyl nonadecanoate). The FA in the lipid extracts were quantified using the calibration curve of the respective standard, except for those in which there was no standard in the FAME mixture. In these cases, the FA with the closest number of carbons and double bonds were used. Thus, the quantification of 16:4*n*-3, 18:4*n*-3, and 20:4*n*-3 was performed using the calibration curve of 20:4*n*-6.

### 2.5. Quantification of Glycolipids and Phospholipids

Glycolipids were quantified by measuring the sugar content using the orcinol method [44]. For that, 100 µg of each dried lipid extract in 2 mL of orcinol solution (0.2% in 70% H_2_SO_4_) were incubated at 80 °C for 20 min. After cooling to room temperature, the absorbance was read at 505 nm (Multiskan GO, Thermo Scientific, Hudson, NH, USA). For determination of the sugar content in each lipid extract, a calibration curve was obtained by performing the same reaction with glucose standards (up to 40 μg) prepared from an aqueous glucose solution (5 mg mL^−1^). The content of glycolipids was estimated by multiplying the sugar content by 2.8 [45].

Phospholipids quantification was achieved by measuring the phosphorous amount using the molybdovanadate method [46], as done in our previous work [44]. The lipid hydrolysis was performed by adding 70% perchloric acid (125 μL) to each dried lipid extract (50 µg in a glass tube washed with 5% nitric acid), followed by incubation on a heating block at 180 °C for 1 h. After cooling to room temperature, Milli-Q water (825 μL), ammonium molybdate (2.5 g 100 mL^−1^ in Milli-Q water; 125 μL), and ascorbic acid (0.1 g mL^−1^ in Milli-Q water; 125 μL) were added to each sample, vortexing after each addition. Samples were placed in a water bath at 100 °C for 10 min and then allowed to cool in a cold-water bath. Phosphate standards containing up to 2 μg of phosphorus (P) were prepared from a sodium dihydrogen phosphate dihydrate solution (100 μg mL^−1^ of P), using the same procedure as samples, excluding the heating on the block heater. After reading the absorbance at 797 nm on the microplate UV-Vis spectrophotometer, the content of phospholipids was estimated by multiplying the amount of P by 25 [47].

### 2.6. Analysis of Polar Lipids by HILIC–MS and MS/MS

Polar lipids were analyzed by hydrophilic interaction liquid chromatography-mass spectrometry (HILIC–MS) (Ultimate 3000 Dionex HPLC system and Q-Exactive^®^ hybrid quadrupole Orbitrap^®^ mass spectrometer; Thermo Fisher Scientific, Waltham, MA, USA). The autosampler chamber was kept at 5 °C and the volume of the sample loop was 20 µL. Mobile phase A was acetonitrile:methanol:water 50:25:25 (*v*/*v*/*v*) and mobile phase B was acetonitrile:methanol 60:40 (*v*/*v*), both containing 1 mM ammonium acetate. The gradient program used was as follows: 0–8 min (40% A), 8–15 min (40–60% A), 15–20 min (60% A), 20–25 min (60–40% A), and 25–35 min (40% A). To analyze each polar lipid extract by HILIC–MS, a volume of 5 µL of a solution containing 5 µL of extract (1 mg mL^−1^), 4 µL of a mixture of phospholipid internal standards, and 91 µL of starting eluent mixture was introduced into the Ascentis Si column (15 cm × 1 mm, 3 µm particle size; Sigma-Aldrich, Darmstadt, Germany) with a flow rate of 40 µL min^−1^ and at 30 °C. The amounts of phospholipid standards (Avanti Polar Lipids, Inc., Alabaster, AL, USA) used in each sample were as follows: 0.012 μg of dimyristoyl phosphatidylglycerol, 0.02 μg of dimyristoyl phosphatidylcholine, 0.02 μg of phosphatidylethanolamine, 0.02 μg of sphingomyelin (17:0), 0.02 μg of lysophosphatidylcholine (19:0), 0.02 μg of ceramide (17:0/d18:1), 0.04 μg of dimyristoyl phosphatidylserine, 0.08 μg of tetramyristoyl cardiolipin, 0.08 μg of dipalmitoyl phosphatidylinositol, and 0.08 μg of dimyristoyl phosphatidic acid. The mass spectrometer was operated simultaneously in positive (3.0 kV) and negative (−2.7 kV) modes. The capillary temperature was 250 °C, sheath gas flow 15 U, and auxiliary gas flow 5 U (arbitrary units). In MS/MS experiments, a resolution of 17,500 and AGC target of 1 × 10^5^ were used. The acquisition cycles consisted of one full scan MS and ten data-dependent MS/MS scans, repeated continuously with the dynamic exclusion of 60 s and an intensity threshold of 2 × 10^4^. MS data were acquired with a high resolution of 70,000 and automatic gain control (AGC) target of 1 × 10^6^. MS/MS data were recorded with a resolution of 17,500, AGC target of 1 × 10^5^, and a normalized collision energy ranging between 25, 30, and 35 eV. Data acquisition and visualization were performed using the Xcalibur data system (V3.3, Thermo Fisher Scientific, Waltham, MA, USA).

In the analysis of LC–MS data using MZmine 2.32 software [48], only peaks with an intensity higher than 1 × 10^4^ were considered. The validated peaks were within the typical retention time of the respective lipid class, with a mass error of less than 5 ppm. The identification of the most lipid species was further validated by analysis of the respective MS/MS data. MS/MS data acquired in positive ion mode allowed us to confirm the polar head group identity and the fatty acyl chain(s) of the molecular species belonging to the monogalactosylmonoacylglyceride (MGMG), digalactosylmonoacylglyceride (DGMG), monogalactosyldiacylglyceride (MGDG), and digalactosyldiacylglyceride (DGDG) classes, observed as [M + NH_4_]^+^ ions, and diacylglyceryl-N,N,N-trimethyl homoserine (DGTS) and monoacylglyceryl-N,N,N-trimethyl homoserine (MGTS) classes, observed as [M + H]^+^ ions. The MS/MS spectra of [M + H]^+^ ions of phosphatidylcholine (PC), lysophosphatidylcholine (LPC), phosphatidylethanolamine (PE), and lysophosphatidylethanolamine (LPE) species allowed us to confirm the respective polar head identity, whereas the fatty acyl composition was achieved by the MS/MS fragmentation of [M + CH_3_COO]^−^ ions (for PC and LPC species) and [M − H]^−^ ions (for PE and LPE species). The identification as sulfoquinovosylmonoacylglyceride (SQMG), sulfoquinovosyldiacylglyceride (SQDG), phosphatidylinositol (PI), lysophosphatidylinositol (LPI), phosphatidylglycerol (PG), or lysophosphatidylglycerol (LPG) species and respective fatty acyl chain(s) was achieved by analysis of the MS/MS spectra of [M − H]^−^ ions. All the MS/MS fragmentation patterns characteristic for the polar lipid classes were previously described [39,49,50]. For normalization of the LC–MS data, the integrated peak areas of the extracted ion chromatograms (XIC) were exported from MZmine software, and the peak area of each species was then divided by the peak area of the lipid standard with the closest retention time. The resulting matrix (.xls) with normalized areas of all species was used to study the origin-driven variations at the level of lipid species. To study variations at the level of lipid classes, a new matrix was created considering the sum of normalized areas of all lipid species within each class.

### 2.7. Statistical Analysis

The statistical analysis was done using R version 4.0.3 [51] in Rstudio version 1.3.1 [52]. The R package Metaboanalyst was used for imputation of missing values and log transformation of the data [53]. Principal component analysis (PCA) was done using R built-in function and R package pcaMethods [54]. Mann–Whitney U test was performed using R built-in functions with the Benjamin–Hochberg correction for the false discovery rate (FDR, *q*-value) [55]. Heatmaps were created from autoscaled data with the R package pheatmap [56] using Euclidean (clustering distance) and ward.D (clustering method). All statistics, graphics and boxplots were created using the R packages ggplot2 [57], plyr [58], dplyr [59], tidyr [60], and ggrepel [61].

## 3. Results

### 3.1. Proximate Composition of Ulva spp. Biomass

The freeze-dried samples of *Ulva* spp. from France (FR) and Portugal (PT, *Ulva rigida* in this case) were analyzed for the determination of moisture and ash, proteins, and lipids contents, while carbohydrates and other compounds were estimated by difference (Table 1).

The mean moisture content (expressed as a percentage of freeze-dried sample weight) in *Ulva* spp. was 8.77 ± 0.25% for FR and 4.45 ± 0.47% for PT (*q* < 0.05). The mean ash content (% of dry weight, %DW) of *Ulva* spp. of PT origin was 32.50 ± 0.34 %DW, while that of FR origin was 13.56 ± 0.52 %DW (*q* < 0.05).

The protein content was estimated from the determination of elemental nitrogen (Table 1) using two nitrogen-protein conversion factors: 6.25, a commonly used factor, and the factor 5, proposed as an appropriate factor for seaweed [40]. Using the factor 6.25, the mean protein content of *Ulva* spp. of FR origin was 15.59 ± 0.09 %DW, while of *U. rigida* of PT origin was 11.13 ± 0.31 %DW. Using factor 5, the mean protein content was 12.47 ± 0.08 %DW for FR and 8.90 ± 0.25 %DW for PT samples. Independently of the nitrogen-to-protein conversion factor used, the protein content was significantly higher in the FR samples than in the PT samples (*q* < 0.05).

The mean of the lipid content estimated by gravimetry of the polar lipid extracts was 2.23 ± 0.10 %DW for the FR samples and 1.14 ± 0.12 %DW for the PT samples. The lipid content was significantly higher in the FR samples than in PT samples (*q* < 0.01).

The content of carbohydrates and other compounds (estimated by difference) of *Ulva* spp. of FR samples (68.60 ± 0.56 %DW) was also significantly higher than that of the PT samples (55.23 ± 0.32 %DW) (*q* < 0.05).

### 3.2. Esterified Fatty Acid Profile

The profile of esterified fatty acids (FA) present in *Ulva* spp. samples from two origins (FR and PT) were determined by GC–MS (Table 2 and Supplementary Figure S1). A total of 14 FA were identified and quantified in both groups, including saturated (SFA), monounsaturated (MUFA), and polyunsaturated (PUFA) fatty acids.

**Figure 1:**
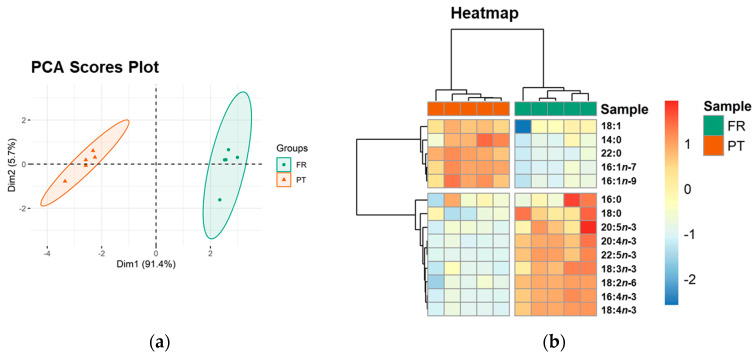
Sample discrimination based on the profile of esterified fatty acids (FA). (**a**) Principal component analysis (PCA) scores plot of the first two dimensions and (**b**) two-dimensional hierarchical cluster heatmap obtained with the FA dataset of *Ulva* spp. from France (FR) and Portugal (PT). Fatty acids are identified as follows: C:D*n*-x (C, number of carbon atoms; D, number of double bonds; x, position of the first double bond relative to the methyl end of the chain).

Multivariate principal component analysis (PCA) of the FA dataset showed that the experimental groups (FR and PT) were well discriminated in a two-dimensional score plot with the first two dimensions (dim), expressing 97.1% of the total variance (91.4% of dim1 and 5.7% of dim2). The two groups were separated along dim1 with FR samples located at positive values and PT samples at negative values (Figure 1a). The most relevant variables explaining the variability of dim1 (contributions > 10%) were PUFA 18:2*n*-6 (41.7%), 16:4*n*-3 (15.5%), 18:4*n*-3 (15.1%), and 22:5*n*-3 (11.5%) (Supplementary Figure S2), which were increased in the FR group.

Univariate analysis was performed using the FA dataset to test for significant differences between the two groups. The Mann–Whitney test revealed significant differences in the content of all FA except SFA 16:0 (Table 2). In addition, the total FA content in the respective polar lipid extract was significantly higher in the FR samples (210.75 ± 24.07 mg g^−1^ ext) than in PT (164.84 ± 11.67 mg g^−1^ ext) (*q* < 0.05).

The clustering of all identified FA was visualized in a two-dimensional hierarchical clustering heatmap (Figure 1b). The FA were divided into two main clusters. From top to bottom, the first cluster included all identified MUFA (18:1, 16:1*n*-7, and 16:1*n*-9) and 2 SFA (14:0 and 22:0), which were significantly more abundant in the PT samples. The second cluster included all identified PUFA and SFA 18:0, which were significantly increased in the FR samples (Table 1).

The atherogenicity index (AI) and thrombogenicity index (TI), proposed as indicators of the propensity of the diet or food to influence the incidence of coronary heart disease (CHD) [37], were calculated from FA profiles. The values of AI and TI were significantly lower in the FR samples, showing a higher content of PUFA. In contrast, the *n*-6/*n*-3 ratio was significantly lower in the PT samples.

### 3.3. Polar Lipidome Profile

The glycolipid and phospholipid contents in polar lipid extracts were estimated by colourimetric methods. The glycolipids represented 464.18 ± 52.45 mg g^−1^ of the PT sample extract and 233.33 ± 11.41 mg g^−1^ of the FR sample extract. The phospholipids represented 89.41 ± 8.88 mg g^−1^ of the PT extract and 27.24 ± 12.50 mg g^−1^ of the FR extract. Glycolipids and phospholipids were significantly higher in PT samples (*q* < 0.01).

A detailed analysis of the polar lipidome of *Ulva* spp. was performed using liquid chromatography coupled with high-resolution mass spectrometry (LC–MS). Considering the two groups (FR and PT), a total of 156 species of polar lipids of distinct molecular weight were identified and semi-quantified (Supplementary Tables S1–S3). The classes of polar lipids identified in both groups were the same, including 6 classes of glycolipids: monogalactosylmonoacylglyceride (MGMG), digalactosylmonoacylglyceride (DGMG), monogalactosyldiacylglyceride (MGDG), digalactosyldiacylglyceride (DGDG), sulfoquinovosylmonoacylglyceride (SQMG), and sulfoquinovosyldiacylglyceride (SQDG); 8 classes of phospholipids: phosphatidylcholine (PC), lysophosphatidylcholine (LPC), phosphatidylethanolamine (PE), lysophosphatidylethanolamine (LPE), phosphatidylinositol (PI), lysophosphatidylinositol (LPI), phosphatidylglycerol (PG), and lysophosphatidylglycerol (LPG); and 2 classes of betaine lipids: diacylglyceryl-N,N,N-trimethyl homoserine (DGTS) and monoacylglyceryl-N,N,N-trimethyl homoserine (MGTS) (Table 3 and Supplementary Figures S3 and S4).

All the lipid species identified in the two *Ulva* spp. were semi-quantified by the corresponding area of the chromatographic peak, divided by the area of the peak of an internal standard. To study the variations in lipid classes, the matrix with the normalized areas of all lipid species was used to calculate the abundance of each lipid class by adding the normalized areas of the lipid species of the same class.

**Figure 2:**
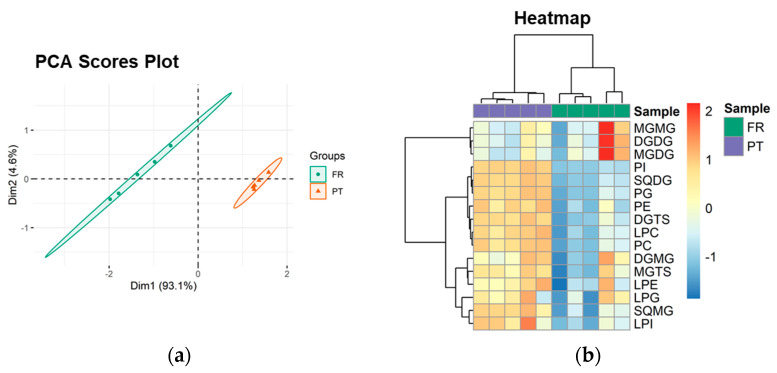
Sample discrimination based on the polar lipid profile at the classes level. (**a**) Principal component analysis (PCA) scores plot of the first two dimensions and (**b**) two-dimensional hierarchical cluster heatmap obtained with the lipid class dataset of *Ulva* spp. from France (FR) and Portugal (PT).

The PCA scores plot of the lipid classes showed that the two groups (PT and FR) were separated in the first two dimensions, and the model captured 97.7% of the total variance in the dataset (93.1% dim1 and 4.6% dim2) (Figure 2a). The two groups were discriminated along dim1, the main discriminating component, with FR samples located at negative values and PT samples located at positive values (Figure 2a). The most relevant contributors to dim1 (contributions > 7%) included PI (35.5%), SQMG (19.1%), SQDG (13.6%), and LPC (7.3%) (Supplementary Figure S5).

Univariate analysis by Mann–Whitney test showed that 9 of the 16 lipid classes (56.3%) were significantly different between the PT and FR samples (*q* < 0.05). DGTS, SQDG, SQMG, LPC, PC, PE, LPI, PG, and PI were increased in the PT samples.

Hierarchical clustering of the lipid class dataset showed the classes divided into two main clusters (dendrogram to the left of the heatmap in Figure 2b). From top to bottom, the first cluster included MGMG, DGDG, and MGDG. The second group consisted of the 9 classes that showed statistically significant differences between the experimental groups and 4 other classes (DGMG, MGTS, LPE, and LPG), all increased in the PT group.

Regarding the lipid species, a distinct number of lipid species was detected between the experimental groups (147 detected in the FR samples and 154 in the PT samples) (Table 3). MGDG (32:0) and MGMG (22:5) have only been identified in the wild *Ulva* spp. of FR origin, while DGMG (14:0), LPE (18:2), SQDG (28:0), SQDG (30:1), SQDG (34:5), SQDG (34:7), SQMG (14:0), SQMG (16:1), and SQMG (18:3) were detected exclusively in farmed *Ulva rigida* of PT (Supplementary Tables S1–S3).

Upon a first visualization of the abundance of lipid species within each class, considerable differences were identified in the abundance of several species, inclusive with the major lipid species distinct by class (Table 3 and Supplementary Figures S6–S8). These variations in lipid species were also assessed using multivariate and univariate statistical analysis.

**Figure 3:**
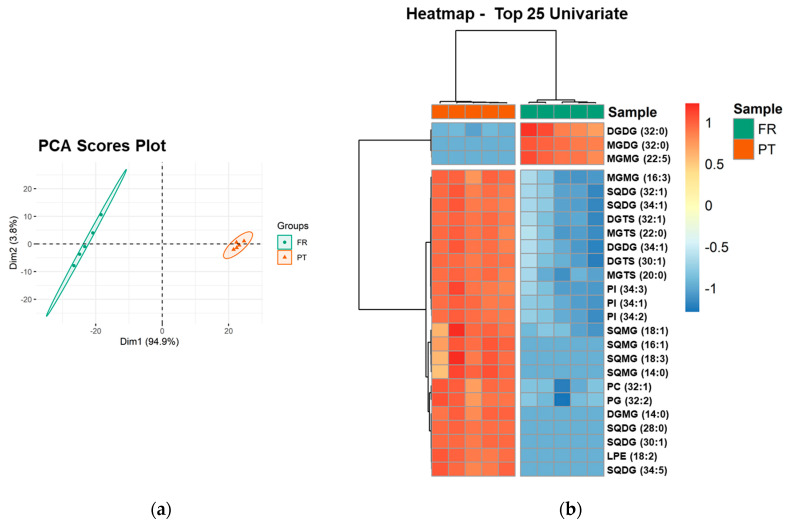
Sample discrimination based on the polar lipid profile at the level of the lipid species. (**a**) Principal component analysis (PCA) scores plot of the first two dimensions obtained with polar lipid species dataset of *Ulva* spp. samples from France (FR) and Portugal (PT). (**b**) Two-dimensional hierarchical cluster heatmap of the top 25 polar lipid species with the lowest *q*-values (*t*-test). Lipid species are labeled as follows: AAAA(C:D) (AAAA, an abbreviation of the lipid class; C, number of carbon atoms in fatty acid(s); D, number of double bonds in fatty acids).

PCA analysis of the lipid species dataset showed that the two groups, the FR and PT samples, were discriminated in a two-dimensional scores plot, expressing 98.7% of the total variance in the dataset (94.9% of dim1 and 3.8% of dim2) (Figure 3a). The most relevant contributors to dim 1 (contributions > 4%) included exclusive species of each group: the galactolipid species MGDG (32:0) and MGMG (22:5), increased in FR samples, and the sulfolipid species SQDG (28:0), SQDG (30:1), and SQMG (16:1), increased in PT samples (Supplementary Figure S9).

Univariate analysis by Mann–Whitney test showed that 128 of 156 lipid species (82.05%) were significantly different between PT and FR samples (*q* < 0.05). A two-dimensional hierarchical clustering heatmap was created using the top 25 lipid species with the lowest *q*-values (Figure 3b). The first level of the upper dendrogram showed the samples clustered into two groups (FR and PT), as observed in the PCA scores plot (Figure 3a). The clustering of the lipid species regarding their similarity in the changes of lipid expression (dendrogram to the left of Figure 3b) showed that they were separated into two main clusters, one comprising three species of neutral glycolipids, DGDG (32:0), MGDG (32:0), and MGMG (22:5), which were more abundant in FR samples. Of these three species, MGDG (32:0) and MGMG (22:5) were only detected in FR samples. The second cluster consisted of 22 lipid species (1 MGMG, 5 SQDG, 2 DGTS, 2 MGTS, 1 DGDG, 3 PI, 4 SQMG, 1 PC, 1 PG, 1 DGMG, and 1 LPE), which were increased in PT samples. Of these 22 species, 10 species (45.45%) had a degree of unsaturation of 1, which is consistent with the higher amount of MUFA found in the PT samples (Table 2). Also, nine species (40.91%) were lysolipid species and eight species (36.36%) were only detected in PT samples.

## 4. Discussion

Macroalgae (seaweed) are natural and sustainable sources of essential nutrients and health-promoting compounds [2,4]. Local environmental conditions and harvest time, among other factors, may have an impact on their composition [4,5,7] and, therefore, on their uses as raw material for applications in food and non-food industries. The present study is the first to initiate a comparison between commercial *Ulva* spp. from different European origins, France (FR, wild-harvested *Ulva* spp.) and Portugal (PT, IMTA-raised *Ulva rigida*), in terms of the proximate composition, esterified fatty acids (FA), and polar lipids, both at the level of the classes and lipid species.

The moisture values (8.77 ± 0.25% for FR and 4.45 ± 0.47% for PT) were within the range of what has already been reported for freeze-dried *Ulva* biomass, 6.41 ± 0.84% [39] and 9.4% [38]. The significant difference in moisture content between the experimental groups (*q* < 0.05) may be related to differences in the lyophilization process and packaging conditions during transport to the laboratory. In terms of ash, proteins, lipids, and carbohydrates and others, the values obtained for FR and PT samples are in the range with those found by other researchers for *Ulva* genus (Supplementary Table S4) [8,16,27,28,29,30,33,39,62,63,64,65,66,67,68,69,70]. However, it is of note that many of these studies used different analytical methodologies for determination of the proximate composition. Beyond the influence of factors as geographical origin, harvest time, species-specific variations, and cultivation protocols [28,29,30,31,32,33,34,35,36,37,38], the variations between different studies may also be related to methodological differences. For example, the lipid content found in most of the studies ranged between 0.5–3% DW [8,27,28,29,30,33,39,62,63,64,67,68,69], but there were reported values up to 13.7% DW [70]. In fact, the high variability in the lipid content reported in the literature may also be related to different methods used, namely different adaptations of Bligh and Dyer and Folch methods or Soxhlet extraction used to obtain lipid extracts.

In this study, PT samples showed significantly higher ash content, while FR samples showed higher content of proteins, lipids, and carbohydrates and other compounds. Regarding the PT samples and comparing studies that used the same methodologies, slightly lower ash values have been previously reported for *U. rigida* from the same origin but harvested in different seasons/years [33,39]. Using the factor 6.25, the protein content obtained from the PT samples was an intermediate value compared to those reported for *U. rigida* from the same origin cultivated in summer 2018 [33] and autumn 2016 [39]. The lipid content obtained from the PT biomass was about two-fold lower than that previously reported for another batch of *Ulva rigida* produced in the same month [29]. A value of carbohydrates and other compounds (estimated by difference) similar to that obtained from PT samples has been reported for Portuguese *U. rigida* produced in autumn 2016 [39] but slightly lower than in summer 2018 [33]. In the case of PT samples, such value (55.23 ± 0.32 %DW) was also similar to the content of insoluble and soluble fibers (55–57 %DW) found in dried (temperature-controlled air-tunnel) and fresh (salted) *Ulva rigida* samples [71]. Regarding the FR samples, the lipid content obtained in this study (2.23 ± 0.10 %DW) was similar to that previously reported (even with variations in the lipid extraction procedures) for *Ulva* species collected in the same region (Brittany coast, France), namely *U. rotundata* collected in December 1991 (1.9 %DW) [62] and *U. armoricana* collected in June 2012 (2.6 %DW) [16].

The differences in the proximate composition of *Ulva* spp. biomass under study (FR and PT) may be related, although not exclusively, to different environmental conditions experienced by different geographic locations. In the case of other Portuguese *U. rigida* biomass farmed in the same location and analyzed for proximate composition using the same methodologies [33,39], the differences can also be related to different environmental conditions experienced by different seasons/years but also to different cultivation protocols. As observed in a laboratory experiment with *U. pertusa* [72], the lipid content increased at low temperature. The higher lipid content found in the FR samples may be related to the fact that they grew in cooler waters. The average water temperature in May in Bretagne (Saint-Malo and Brest, FR) was 12.6 °C (Source: www.seatemperature.org/europe accessed on 12 November 2020), while that recorded in May 2017 in seaweed tanks of ALGAplus (Aveiro, PT) was 17.5 °C [29]. In addition to temperature, the possible effect of other environmental factors, namely salinity, light exposure, and nutrient availability, on the lipid content cannot be excluded [72]. Probably, wild *Ulva* spp. (FR) also experienced stronger water currents than *Ulva rigida* cultivated in IMTA (PT). *Ulva* spp. from FR were subjected to tidal variations, while *Ulva rigida* cultivated under IMTA conditions (PT) grew in a more constant environment not limited by nutrients. However, the chosen cultivation strategy in these aquaculture systems also impacts nutritional composition, as the focus on the increase of the biomass yield is normally detrimental to nitrogen accumulation, and thus to the protein content [73]. On other hand, the possibility of having more than a single *Ulva* species cannot be excluded for wild-harvested biomass (FR), as well as of having species-specific variations [34,63]. More studies under controlled conditions are necessary to dissect the effect of each environmental factor but also of intrinsic factors (life cycle and phylogeny) and cultivation/processing protocols on the composition of *Ulva* spp., particularly in terms of lipid content and its detailed composition.

Despite the variations in proximate composition, the same esterified fatty acids (FA), as well as the same classes of polar lipids were identified from lipid extracts of *Ulva* spp. of different origins (FR and PT). However, the profiles of FA and polar lipids (the latter at level of the classes and lipid species) differed significantly. 

Overall, the results of the FA analysis are in line with those presented in other studies (Supplementary Table S4), showing high levels of SFA 16:0 and C16 and C18 PUFA in *Ulva* spp. [8,16,29,30,32,34,62,67]. The amount of MUFA 18:1 was calculated from the sum of two peaks, which should correspond to *n*-7 and *n*-9, as shown in previous studies [30,31,32,34]. When comparing the FA profile obtained in this study with other previously published, it is noteworthy that most of the studies used different methodologies for lipid extraction and fatty acid analysis, even considering only the analysis of FAME derivatives (Supplementary Table S4). The differences reported for the FA profile within the same genus may also be related to different methods used, not excluding the influence of environmental and other factors mentioned above.

Comparing the experimental groups under study, the FR samples showed a higher content of PUFA, which are recognized protective dietary factors against coronary heart disease (CHD) [74]. In contrast, the *n*-6/*n*-3 ratio was significantly lower in the PT samples. However, for both groups, *n*-6/*n*-3 ratio was less than 1, which is considered beneficial for human health not only in reducing CHD [10] but also other pathologies such as cancer and associated complications [75,76].

Regarding the effect of temperature changes on algae, it is well accepted that the unsaturation degree increases at lower temperatures [77]. Such effect may have contributed to the higher PUFA content found in the FR samples grown in cooler water. However, other environmental factors (e.g., light, salinity, and nutrients) can modulate the FA profile [72]. Also, the influence of non-environmental factors in the FA profile, namely species-specific variations [34,63], cannot be excluded. On other hand, the differences in the composition of PUFA between FR and PT samples may lead to an altered production of oxylipins formed by enzymatic oxidation, which deserves to be explored in future studies. A previous work with *Ulva* species collected at various sampling sites in the lagoon Ria Formosa (Faro, Portugal) showed that the production of polyunsaturated aldehydes is dependent on the PUFA content [78]. This is important when selecting *Ulva* biomass for further applications, namely in the food industry, as this class of substances results in different flavors.

The classes of polar lipids identified in the two groups (FR and PT) are in agreement with those previously described for Portuguese *Ulva rigida* [29,33,39,79]. Among these classes showing significant variations, sulfolipids are the most recognized as bioactive compounds [11,12,13].

The total number of lipid species identified from FR samples (147) was lower than that identified in PT samples (154). SQDG (28:0) and SQDG (30:1), detected only in PT samples, were between site-specific lipid species previously identified in wild *Ulva* spp. from the Iberian Atlantic coast and farmed *Ulva rigida* from Aveiro (PT) with the potential to trace their geographic origin [33].

Despite the differences found in this work at level of the lipid species, it is of note that both batches (PT and FR) showed polar lipid species that were previously identified as contributing up to 50% similarity of the *Ulva rigida* cultivated in different seasons/years [79]. Effectively, a part of the lipid signature seems to remain conserved in *Ulva* species. Nonetheless, the strength of the results presented in this study is limited, namely by the single sampling. In further studies, it should be interesting to evaluate if the differences found at the level of lipid species between FR and PT samples remain in other samplings at different months and years. Additionally, more work is needed to understand the influence of the different environmental and non-environmental factors on the variations observed and then to assess the impact on potential applications.

Regarding the interest of polar lipids as health-promoting compounds, glycolipid species with previously reported bioactivity have been identified in *Ulva* spp. samples of FR and PT, namely SQDG (16:0/16:0) [12] and SQMG (16:0) [13] with antimicrobial activity and MGDG (18:4/16:4) [14], MGMG (16:3) [15], and MGMG (16:2) [15] with anti-inflammatory activity. Of note, MGMG (16:3) was in the top 25 lipid species with the most significant variation between FR and PT samples. In contrast, no significant variation was observed in the abundance of MGMG (16:2). Although less studied in terms of bioactive properties compared to glycolipids, phospholipids and betaine lipids of algae have also been recognized as important bioactive compounds; namely, the PG and PC species of the red macroalga *Palmaria palmata* [80] and DGTS species isolated from the microalga *Nannochloropsis granulata* [81] have been identified as potential anti-inflammatory agents. Further studies are needed to explore the biological activity of *Ulva* spp. polar lipids and monitor the biomass value of *Ulva* species based on their location and time of harvesting but also on other factors as species-specific differences, cultivation conditions, and processing protocols. The presence of such bioactive polar lipids is important for the valorization of *Ulva* spp. as a functional food, especially in view of the global need for healthy and sustainable diets.

## 5. Conclusions

The commercial biomass of *Ulva* may differ considerably in terms of their composition at a given time. In this study, commercial *Ulva* spp. harvested in May of the same year from different European origins were compared: wild-harvested *Ulva* spp. from France (FR) and farm-raised *Ulva rigida* from Portugal (PT). As supported by statistical analysis, the PT samples showed higher ash content, while the FR samples revealed higher levels of proteins, lipids, and carbohydrates and other compounds. The profile of esterified fatty acids (FA), as well as of polar lipids containing esterified FA at the level of lipid classes and lipid species, were also significantly different. The polar lipid species that showed significant variations included potentially bioactive lipids, namely neutral glycolipids as MGMG (16:3), MGDG (18:4/16:4), MGMG (16:3), and sulfolipids as SQDG (16:0/16:0) and SQMG (16:0). 

Improved knowledge on the composition and lipid variation of commercial *Ulva* spp. will allow us to optimize the best uses of these green macroalgae and valorize specific production sites. This is particularly relevant given the importance of *Ulva* spp. as a functional food, contributing to the promotion of a healthy and sustainable diet.

## Figures and Tables

**Table 1:** Elemental and proximate composition of *Ulva* spp. samples from France (FR) and Portugal (PT), expressed as a percentage of the dry weight (%DW), excluding moisture (% of freeze-dried sample weight) ^1^.

	FR	PT	Statistical Significance
**Elemental composition**			
C (%DW)	34.12 ± 0.22	25.97 ± 0.76	**
H (%DW)	6.13 ± 0.21	2.82 ± 0.08	**
N (%DW)	2.49 ± 0.02	1.78 ± 0.05	**
S (%DW)	2.09 ± 0.37	8.07 ± 0.52	**
**Proximate composition**			
Moisture (%)	8.77 ± 0.25	4.45 ± 0.47	*
Ash (%DW)	13.56 ± 0.52	32.50 ± 0.34	*
Proteins (%DW)			
N × 6.25	15.59 ± 0.09	11.13 ± 0.31	*
N × 5	12.47 ± 0.08	8.90 ± 0.25	*
Lipids (%DW)	2.23 ± 0.10	1.14 ± 0.12	**
Carbohydrates and others (%DW)	68.60 ± 0.56	55.23 ± 0.32	*

^1^ Values are means ± standard deviations for five replicates (*n* = 5). Carbohydrates and others were estimated by the difference of ash, proteins (N × 6.25) and lipids. Statistical significance among the experimental groups was assessed by performing the Mann–Whitney test (*, *q* < 0.05; **, *q* < 0.01).

**Table 2:** Profile of esterified fatty acids present in *Ulva* spp. samples from France (FR) and Portugal (PT), determined by gas chromatography-mass spectrometry (GC–MS) and expressed in mg g^−1^ of polar lipid extract (ext) ^1^.

	FR (mg g^−1^ Ext)	PT (mg g^−1^ Ext)	Statistical Significance
**Saturated fatty acids (SFA)**	98.37 ± 9.74	88.40 ± 5.35	NS
14:0	5.14 ± 0.19	6.00 ± 0.41	*
16:0	75.70 ± 8.97	69.94 ± 6.03	NS
18:0	13.63 ± 3.08	7.05 ± 1.38	**
22:0	3.90 ± 0.11	5.41 ± 0.17	**
**Monounsaturated fatty acids (MUFA)**	27.23 ± 7.03	48.89 ± 4.42	*
16:1*n*-7	3.98 ± 0.33	8.29 ± 0.77	**
16:1*n*-9	3.75 ± 0.23	6.21 ± 0.62	**
18:1	19.51 ± 6.57	34.39 ± 3.08	**
**Polyunsaturated fatty acids (PUFA)**	85.14 ± 9.86	27.55 ± 2.07	*
16:4*n*-3	21.43 ± 3.23	5.10 ± 0.44	**
18:2*n*-6	8.52 ± 1.08	0.91 ± 0.30	**
18:3*n*-3	13.29 ± 1.41	8.50 ± 0.74	**
18:4*n*-3	28.57 ± 3.68	6.91 ± 0.58	**
20:4*n*-3	2.92 ± 0.21	1.75 ± 0.04	**
20:5*n*-3	2.66 ±0.24	2.17 ± 0.06	**
22:5*n*-3	7.75 ± 1.14	2.21 ± 0.04	**
**Total PUFA *n*-3**	76.62 ± 8.79	26.64 ± 1.86	*
**PUFA *n*-6/ PUFA *n*-3**	0.11 ± 0.00	0.03 ± 0.01	*
**Total FA**	210.75 ± 24.07	164.84 ± 11.67	*
**AI**	0.87 ± 0.10	1.23 ± 0.02	*
**TI**	0.37 ± 0.04	0.60 ± 0.07	*

^1^ Values are means ± standard deviations for five replicates (corresponding to five lipid extracts, *n* = 5). Fatty acids (FA) are identified as follows: C:D*n*-x (C, number of carbon atoms; D, number of double bonds; x, position of the first double bond relative to the methyl end of the chain). The statistical significance among the experimental groups was assessed by performing the Mann–Whitney test (*, *q* < 0.05; **, *q* < 0.01; NS, not significant). Other abbreviations: AI, atherogenicity index; TI, thrombogenicity index.

**Table 3:** Summary of polar lipid classes, total number of lipid species, and major lipid species in each lipid class identified by hydrophilic interaction liquid chromatography-mass spectrometry (HILIC–MS) in polar lipid extracts of *Ulva* spp. of France (FR) and Portugal (PT) ^1^.

Polar Lipid Classes	Number of Lipid Species		Major Lipid Species by Class	
	FR	PT	FR	PT
**Glycolipids**	47	53		
MGMG	7	6	MGMG (16:0)	MGMG (16:4)
DGMG	4	5	DGMG (16:0)	DGMG (16:0)
MGDG	9	8	MGDG (34:8)	MGDG (34:8)
DGDG	12	12	DGDG (32:0)	DGDG (34:1)
SQMG	2	5	SQMG (16:0)	SQMG (16:0)
SQDG	13	17	SQDG (34:1)	SQDG (34:1)
**Phospholipids**	49	50		
PC	12	12	PC (34:1)	PC (36:2)
LPC	9	9	LPC (16:0)	LPC (16:0)
PE	6	6	PE (32:1)	PE (32:1)
LPE	5	6	LPE (16:0)	LPE (16:1)
PI	3	3	PI (34:1)	PI (34:1)
LPI	1	1	LPI (16:0)	LPI (16:0)
PG	9	9	PG (34:4)	PG (34:4)
LPG	4	4	LPG (16:1)	LPG (16:1)
**Betaine lipids**	51	51		
DGTS	36	36	DGTS (34:4)	DGTS (32:1)
MGTS	15	15	MGTS (18:3)	MGTS (16:0)
**Total**	147	154		

^1^ Lipid species are identified as follows: AAAA(C:D) (AAAA, an abbreviation of the lipid class; C, number of carbon atoms in fatty acid(s); D, number of double bonds in fatty acids). Abbreviations of the lipid classes: DGDG, digalactosyldiacylglyceride; DGMG, digalactosylmonoacylglyceride; DGTS, diacylglyceryl-N,N,N-trimethyl homoserine; MGDG, monogalactosyldiacylglyceride; MGMG, monogalactosylmonoacylglyceride; MGTS, monoacylglyceryl-N,N,N-trimethyl homoserine; LPC, lysophosphatidylcholine; LPE, lysophosphatidylethanolamine; LPG, phosphatidylglycerol; LPI, lysophosphatidylinositol; PC, phosphatidylcholine; PE, phosphatidylethanolamine; PG, phosphatidylglycerol; PI, phosphatidylinositol; SQDG, sulfoquinovosyldiacylglyceride; SQMG, sulfoquinovosylmonoacylglyceride.

## Supplementary Materials

The following are available online at https://www.mdpi.com/article/10.3390/foods10050914/s1, Table S1: Glycolipids identified by LC-MS and MS/MS of *Ulva* spp. samples (mass error < 5 ppm); Table S2: Phospholipids identified by LC-MS and MS/MS of *Ulva* spp. samples (mass error < 5 ppm); Table S3: Betaine lipids identified by LC-MS and MS/MS of *Ulva* spp. samples (mass error < 5 ppm); Table S4: Data on the proximate composition and fatty acid profile found for *Ulva* genus in this study and other published studies; Figure S1: Representative examples of total ion chromatograms (TIC) obtained by GC-MS; Figure S2: Variables ordered by contribution (%) to dimension (dim) 1 of the principal component analysis (PCA) of esterified fatty acids dataset; Figure S3: Representative examples of total ion chromatograms (TIC) obtained by analysis of the lipid standards and the polar lipids extracts of *Ulva* spp. from Portugal (PT) and France (FR), acquired on positive mode (+) and negative mode (–); Figure S4: Extracted ion chromatograms (XIC) obtained by LC-MS analysis of the lipid standards, acquired on positive mode (+) and negative mode (–); Figure S5: Variables ordered by contribution (%) to dimension (dim) 1 of the principal component analysis (PCA) of lipid classes dataset; Figure S6: Abundance (normalized XIC area) of glycolipid species identified by LC-MS and MS/MS of *Ulva* spp. samples from France (FR) and Portugal (PT), presented by class: (a) DGDG, (b) MGDG, (c) DGMG, (d) MGMG, (e) SQDG, and (f) SQMG species; Figure S7: Abundance (normalized XIC area) of phospholipid species identified by LC-MS and MS/MS of *Ulva* spp. samples from France (FR) and Portugal (PT), presented by class: (a) PC, (b) PE, (c) LPC, (d) LPE, (e) PG, (f) LPG, and (g) PI species; Figure S8: Abundance (normalized area) of betaine lipid species identified by LC-MS and MS/MS of *Ulva* spp. samples from France (FR) and Portugal (PT), presented by class: (a) DGTS and (b) MGTS species; Figure S9: Variables ordered by contribution (%) to dimension (dim) 1 of the principal component analysis (PCA) of lipid species dataset.

## References

[1] FAO—Food and Agricultural Organization of the United Nations, WHO—World Health Organization (2019). Sustainable Healthy Diets—Guiding Principles.

[2] Tanna B., Mishra A. (2018). Metabolites unravel nutraceutical potential of edible seaweeds: An emerging source of functional food. Compr. Rev. Food Sci. Food Saf..

[3] Cherry P., O’Hara C., Magee P.J., McSorley E.M., Allsopp P.J. (2019). Risks and benefits of consuming edible seaweeds. Nutr. Rev..

[4] Stengel D.B., Connan S., Popper Z.A. (2011). Algal chemodiversity and bioactivity: Sources of natural variability and implications for commercial application. Biotechnol. Adv..

[5] Li-Beisson Y., Thelen J.J., Fedosejevs E., Harwood J.L. (2019). The lipid biochemistry of eukaryotic algae. Prog. Lipid Res..

[6] Eismann A.I., Perpetuo Reis R., Ferreira da Silva A., Negrão Cavalcanti D. (2020). *Ulva* spp. carotenoids: Responses to environmental conditions. Algal Res..

[7] Guschina I.A., Harwood J.L., Kainz M., Brett M.T., Arts M.T. (2009). Algal lipids and effect of the environment on their biochemistry. Lipids in Aquatic Ecosystems.

[8] Gosch B.J., Magnusson M., Paul N.A., de Nys R. (2012). Total lipid and fatty acid composition of seaweeds for the selection of species for oil-based biofuel and bioproducts. GCB Bioenergy.

[9] Swanson D., Block R., Mousa S.A. (2012). Omega-3 fatty acids EPA and DHA: Health benefits throughout life. Adv. Nutr..

[10] Sakamoto A., Saotome M., Iguchi K., Maekawa Y. (2019). Marine-derived omega-3 polyunsaturated fatty acids and heart failure: Current understanding for basic to clinical relevance. Int. J. Mol. Sci..

[11] Da Costa E., Silva J., Mendonça S.H., Abreu M.H., Domingues M.R. (2016). Lipidomic approaches towards deciphering glycolipids from microalgae as a reservoir of bioactive lipids. Mar. Drugs.

[12] Wang H., Li Y.-L., Shen W.-Z., Rui W., Ma X.-J., Cen Y.-Z. (2007). Antiviral activity of a sulfoquinovosyldiacylglycerol (SQDG) compound isolated from the green alga *Caulerpa racemosa*. Bot. Mar..

[13] Arunkumar K., Selvapalam N., Rengasamy R. (2005). The antibacterial compound sulphoglycerolipid 1-0 palmitoyl-3-0(6′-sulpho-α-quinovopyranosyl)-glycerol from *Sargassum wightii* Greville (Phaeophyceae). Bot. Mar..

[14] Banskota A.H., Gallant P., Stefanova R., Melanson R., O’Leary S.J. (2013). Monogalactosyldiacylglycerols, potent nitric oxide inhibitors from the marine microalga *Tetraselmis chui*. Nat. Prod. Res..

[15] Banskota A.H., Stefanova R., Gallant P., Osborne J.A., Melanson R., O’Leary S.J. (2013). Nitric oxide inhibitory activity of monogalactosylmonoacylglycerols from a freshwater microalgae *Chlorella sorokiniana*. Nat. Prod. Res..

[16] Kendel M., Wielgosz-Collin G., Bertrand S., Roussakis C., Bourgougnon N., Bedoux G. (2015). Lipid composition, fatty acids and sterols in the seaweeds *Ulva armoricana*, and *Solieria chordalis* from Brittany (France): An analysis from nutritional, chemotaxonomic, and antiproliferative activity perspectives. Mar. Drugs.

[17] Plouguerné E., da Gama B.A.P., Pereira R.C., Barreto-Bergter E. (2014). Glycolipids from seaweeds and their potential biotechnological applications. Front. Cell Infect. Microbiol..

[18] Cotas J., Leandro A., Pacheco D., Gonçalves A.M.M., Pereira L. (2020). A comprehensive review of the nutraceutical and therapeutic applications of red seaweeds (Rhodophyta). Life.

[19] Gullón B., Gagaoua M., Barba F.J., Gullón P., Zhang W., Lorenzo J.M. (2020). Seaweeds as promising resource of bioactive compounds: Overview of novel extraction strategies and design of tailored meat products. Trends Food Sci. Technol..

[20] Thiyagarasaiyar K., Goh B.-H., Jeon Y.-J., Yow Y.-Y. (2020). Algae metabolites in cosmeceutical: An overview of current applications and challenges. Mar. Drugs.

[21] Ścieszka S., Klewicka E. (2019). Algae in food: A general review. Crit. Rev. Food Sci. Nutr..

[22] Brown E.S., Allsopp P.J., Magee P.J., Gill C.I., Nitecki S., Strain C.R., McSorley E.M. (2014). Seaweed and human health. Nutr. Rev..

[23] FAO—Food and Agricultural Organization of the United Nations (2018). The Global Status of Seaweed Production, Trade and Utilization.

[24] Mantri V.A., Kazi M.A., Balar N.B., Gupta V., Gajaria T. (2020). Concise review of green algal genus *Ulva* Linnaeus. J. Appl. Phycol..

[25] Bolton J.J., Cyrus M.D., Brand M.J., Joubert M., Macey B.M. (2016). Why grow *Ulva*? Its potential role in the future of aquaculture. Perspect. Phycol..

[26] Hafting J.T., Critchley A.T., Cornish M.L., Hubley S.A., Archibald A.F. (2012). On-land cultivation of functional seaweed products for human usage. J. Appl. Phycol..

[27] Gadberry B.A., Colt J., Maynard D., Boratyn D.C., Webb K., Johnson R.B., Saunders G.W., Boyer R.H. (2018). Intensive land-based production of red and green macroalgae for human consumption in the Pacific Northwest: An evaluation of seasonal growth, yield, nutritional composition, and contaminant levels. Algae.

[28] Mohy El-Din S.M. (2019). Temporal variation in chemical composition of *Ulva lactuca* and *Corallina mediterranea*. Int. J. Environ. Sci. Technol..

[29] Moreira A.S.P., da Costa E., Melo T., Sulpice R., Cardoso S.M., Pitarma B., Pereira R., Abreu M.H., Domingues P., Calado R. (2020). Seasonal plasticity of the polar lipidome of *Ulva rigida* cultivated in a sustainable integrated multi-trophic aquaculture. Algal Res..

[30] Nelson M.M., Phleger C.F., Nichols P.D. (2002). Seasonal lipid composition in macroalgae of the northeastern pacific ocean. Bot. Mar..

[31] Sanina N.M., Goncharova S.N., Kostetsky E.Y. (2008). Seasonal changes of fatty acid composition and thermotropic behavior of polar lipids from marine macrophytes. Phytochemistry.

[32] Serviere-Zaragoza E., Hurtado M.A., Manzano-Sarabia M., Mazariegos-Villarreal A., Reza M., Arjona O., Palacios E. (2015). Seasonal and interannual variation of fatty acids in macrophytes from the Pacific coast of Baja California Peninsula (Mexico). J. Appl. Phycol..

[33] Da Costa E., Ricardo F., Melo T., Mamede R., Abreu M.H., Domingues P., Domingues M.R., Calado R. (2020). Site-specific lipidomic signatures of sea lettuce (*Ulva* spp., Chlorophyta) hold the potential to trace their geographic origin. Biomolecules.

[34] Cardoso C., Ripol A., Afonso C., Freire M., Varela J., Quental-Ferreira H., Pousão-Ferreira P., Bandarra N. (2017). Fatty acid profiles of the main lipid classes of green seaweeds from fish pond aquaculture. Food Sci. Nutr..

[35] Alsufyani T., Califano G., Deicke M., Grueneberg J., Weiss A., Engelen A.H., Kwantes M., Mohr J.F., Ulrich J.F., Wichard T. (2020). Macroalgal–bacterial interactions: Identification and role of thallusin in morphogenesis of the seaweed *Ulva* (Chlorophyta). J. Exp. Bot..

[36] Hoxmark R.C. (1976). Protein composition of different stages in the life cycle of *Ulva mutabilis*, Føyn. Planta.

[37] Msuya F.E., Neori A. (2008). Effect of water aeration and nutrient load level on biomass yield, N uptake and protein content of the seaweed *Ulva lactuca* cultured in seawater tanks. J. Appl. Phycol..

[38] Silva A.F.R., Abreu H., Silva A.M.S., Cardoso S.M. (2019). Effect of oven-drying on the recovery of valuable compounds from *Ulva rigida*, *Gracilaria* sp. and *Fucus vesiculosus*. Mar. Drugs.

[39] Lopes D., Moreira A.S.P., Rey F., da Costa E., Melo T., Maciel E., Rego A., Abreu M.H., Domingues P., Calado R. (2019). Lipidomic signature of the green macroalgae *Ulva rigida* farmed in a sustainable integrated multi-trophic aquaculture. J. Appl. Phycol..

[40] Angell A.R., Mata L., de Nys R., Paul N.A. (2016). The protein content of seaweeds: A universal nitrogen-to-protein conversion factor of five. J. Appl. Phycol..

[41] Bligh E.G., Dyer W.J. (1959). A rapid method of total lipid extraction and purification. Can. J. Biochem. Phys..

[42] Aued-Pimentel S., Lago J.H.G., Chaves M.H., Kumagai E.E. (2004). Evaluation of a methylation procedure to determine cyclopropenoids fatty acids from *Sterculia striata* St. Hil. Et Nauds seed oil. J. Chromatogr. A.

[43] Christie W.W. The Lipid Web. https://www.lipidmaps.org/resources/lipidweb/index.php?page=ms/methesters/me-arch/index.htm.

[44] Da Costa E., Melo T., Moreira A.S.P., Bernardo C., Helguero L., Ferreira I., Cruz M.T., Rego A.M., Domingues P., Calado R. (2017). Valorization of lipids from *Gracilaria* sp. through lipidomics and decoding of antiproliferative and anti-inflammatory activity. Mar. Drugs.

[45] Bell B.M., Daniels D.G.H., Fearn T., Stewart B.A. (1987). Lipid compositions, baking qualities and other characteristics of wheat varieties grown in the U.K. J. Cereal Sci..

[46] Bartlett E.M., Lewis D.H. (1970). Spectrophotometric determination of phosphate esters in the presence and absence of orthophosphate. Anal. Biochem..

[47] Chapman G.W. (1980). A conversion factor to determine phospholipid content in soybean and sunflower crude oils. J. Am. Oil Chem. Soc..

[48] Pluskal T., Castillo S., Villar-Briones A., Orešič M. (2010). MZmine 2: Modular framework for processing, visualizing, and analyzing mass spectrometry-based molecular profile data. BMC Bioinform..

[49] Monteiro J.P., Rey F., Melo T., Moreira A.S.P., Arbona J.-F., Skjermo J., Forbord S., Funderud J., Raposo D., Kerrison P.D. (2020). The unique lipidomic signatures of *Saccharina latissima* can be used to pinpoint their geographic origin. Biomolecules.

[50] Da Costa E., Amaro H.M., Melo T., Guedes A.C., Domingues M.R. (2020). Screening for polar lipids, antioxidant, and anti-inflammatory activities of *Gloeothece* sp. lipid extracts pursuing new phytochemicals from cyanobacteria. J. Appl. Phycol..

[51] R Core Team R: A Language and Environment for Statistical Computing. https://www.R-project.org/.

[52] RStudio Team RStudio: Integrated Development Environment for R. https://www.rstudio.com/.

[53] Xia J., Wishart D.S. (2016). Using MetaboAnalyst 3.0 for comprehensive metabolomics data analysis. Curr. Protoc. Bioinform..

[54] Stacklies W., Redestig H., Scholz M., Walther D., Selbig J. (2007). pcaMethods--A bioconductor package providing PCA methods for incomplete data. Bioinformatics.

[55] Benjamini Y., Hochberg Y. (1995). Controlling the false discovery rate: A practical and powerful approach to multiple testing. J. Roy. Stat. Soc. Series B Methodol..

[56] Kolde R. Pheatmap: Pretty Heatmaps. https://rdrr.io/cran/pheatmap/.

[57] Wickham H., Chang W., Henry L., Pedersen T.L., Takahashi K., Wilke C., Woo K., Yutani H. *ggplot2*—Elegant Graphics for Data Analysis. https://ggplot2.tidyverse.org/.

[58] Wickham H. (2011). The split-apply-combine strategy for data analysis. J. Stat. Softw..

[59] Wickham H., François R., Henry L., Müller K. dplyr: A Grammar of Data Manipulation. https://rdrr.io/cran/dplyr/.

[60] Wickham H., Henry L. tidyr: Easily Tidy Data with ’Spread()’ and ’Gather()’ Functions. https://rdrr.io/cran/tidyr/.

[61] Slowikowski K., Schep A., Hughes S., Lukauskas S., Irisson J.-O., Kamvar Z.N., Ryan T., Christophe D., Hiroaki Y., Gramme P. ggrepel: Automatically Position Non-Overlapping Text Labels with ’ggplot2’. https://rdrr.io/cran/ggrepel/.

[62] Fleurence J., Gutbier G., Mabeau S., Leray C. (1994). Fatty acids from 11 marine macroalgae of the French Brittany coast. J. Appl. Phycol..

[63] Kumari P., Kumar M., Gupta V., Reddy C.R.K., Jha B. (2010). Tropical marine macroalgae as potential sources of nutritionally important PUFAs. Food Chem..

[64] Van Ginneken V.J., Helsper J.P., de Visser W., van Keulen H., Brandenburg W.A. (2011). Polyunsaturated fatty acids in various macroalgal species from North Atlantic and tropical seas. Lipids Health Dis..

[65] Yaich H., Garna H., Besbes S., Paquot M., Blecker C., Attia H. (2011). Chemical composition and functional properties of *Ulva lactuca* seaweed collected in Tunisia. Food Chem..

[66] Khairy H.M., El-Shafay S.M. (2013). Seasonal variations in the biochemical composition of some common seaweed species from the coast of Abu Qir Bay, Alexandria, Egypt. Oceanologia.

[67] Maehre H.K., Malde M.K., Eilertsen K.E., Elvevoll E.O. (2014). Characterization of protein, lipid and mineral contents in common Norwegian seaweeds and evaluation of their potential as food and feed. J. Sci. Food Agric..

[68] Paiva L., Lima E., Neto A.I., Marcone M., Baptista J. (2016). Health-promoting ingredients from four selected Azorean macroalgae. Food Res. Int..

[69] Neto R.T., Marçal C., Queirós A.S., Abreu H., Silva A.M.S., Cardoso S.M. (2018). Screening of *Ulva rigida*, *Gracilaria* sp., *Fucus vesiculosus* and *Saccharina latissima* as functional ingredients. Int. J. Mol. Sci..

[70] Roleda M.Y., Lage S., Aluwini D.F., Rebours C., Brurberg M.B., Nitschke U., Gentili F.G. (2021). Chemical profiling of the Arctic sea lettuce *Ulva lactuca* (Chlorophyta) mass-cultivated on land under controlled conditions for food applications. Food Chem..

[71] Pinheiro V.F., Marçal C., Abreu H., Lopes da Silva J.A., Silva A.M.S., Cardoso S.M. (2019). Physicochemical changes of air-dried and salt-processed *Ulva rigida* over storage time. Molecules.

[72] Floreto E.A.T., Hirata H., Ando S., Yamasaki S. (1993). Effects of temperature, light intensity, salinity and source of nitrogen on the growth, total lipid and fatty acid composition of *Ulva pertusa* Kjellman (Chlorophyta). Bot. Mar..

[73] Abreu M.H., Pereira R., Yarish C., Buschmann A.H., Sousa-Pinto I. (2011). IMTA with *Gracilaria vermiculophylla*: Productivity and nutrient removal performance of the seaweed in a land-based pilot scale system. Aquaculture.

[74] Ulbricht T.L.V., Southgate D.A.T. (1991). Coronary heart disease: Seven dietary factors. Lancet.

[75] Dimri M., Bommi P.V., Sahasrabuddhe A.A., Khandekar J.D., Dimri G.P. (2010). Dietary omega-3 polyunsaturated fatty acids suppress expression of EZH2 in breast cancer cells. Carcinogenesis.

[76] Freitas R.D.S., Campos M.M. (2019). Protective effects of omega-3 fatty acids in cancer-related complications. Nutrients.

[77] Thompson G.A. (1996). Lipids and membrane function in green algae. Biochim. Biophys. Acta Lipids Lipid Metab..

[78] Alsufyani T., Engelen A.H., Diekmann O.E., Kuegler S., Wichard T. (2014). Prevalence and mechanism of polyunsaturated aldehydes production in the green tide forming macroalgal genus *Ulva* (Ulvales, Chlorophyta). Chem. Phys. Lipids.

[79] Lopes D., Melo T., Rey F., Costa E., Moreira A.S.P., Abreu M.H., Domingues P., Lillebø A.I., Calado R., Rosário Domingues M. (2021). Insights of species-specific polar lipidome signatures of seaweeds fostering their valorization in the blue bioeconomy. Algal Res..

[80] Banskota A.H., Stefanova R., Sperker S., Lall S.P., Craigie J.S., Hafting J.T., Critchley A.T. (2014). Polar lipids from the marine macroalga *Palmaria palmata* inhibit lipopolysaccharide-induced nitric oxide production in RAW264.7 macrophage cells. Phytochemistry.

[81] Banskota A.H., Stefanova R., Sperker S., McGinn P.J. (2013). New diacylglyceryltrimethylhomoserines from the marine microalga *Nannochloropsis granulata* and their nitric oxide inhibitory activity. J. Appl. Phycol..

