# Supplementary material for: Polar Lipids of Commercial Ulva spp. of Different Origins: Profiling and Relevance for Seaweed Valorization
Source: Foods. 2021 Apr 21;10(5):914. doi: 10.3390/foods10050914 (PMC8143280; doi:10.3390/foods10050914)
Supplement: Supplementary file 1 [file foods-10-00914-s001.zip › foods-1176686-supplementary.pdf]

Supplementary material

Article

Polar lipids of commercial *Ulva* spp. of different origins: profiling and relevance for seaweed valorization

Ana S. P. Moreira[1,2,*], Elisabete da Costa[2,3], Tânia Melo[2,3], Diana Lopes[2,3], Adriana C. S. Pais[1], Sónia A. O. Santos[1], Bárbara Pitarma[4], Madalena Mendes[4,5], Maria H. Abreu[4], Pi Nyvall Collén[6], Pedro Domingues[2], M. Rosário Domingues[2,3]

[1] CICECO - Aveiro Institute of Materials, Department of Chemistry, Santiago University Campus, University of Aveiro, 3810-193 Aveiro, Portugal; ana.moreira@ua.pt; a.c.p.s@ua.pt; santos.sonia@ua.pt
[2] LAQV-REQUIMTE, Department of Chemistry, Santiago University Campus, University of Aveiro, 3810-193 Aveiro, Portugal; p.domingues@ua.pt
[3] CESAM - Centre for Environmental and Marine Studies, Department of Chemistry, Santiago University Campus, University of Aveiro, 3810-193 Aveiro, Portugal; elisabetecosta@ua.pt; taniamelo@ua.pt; dianasalzedaslopes@ua.pt; mrd@ua.pt
[4] ALGAplus - Produção e Comercialização de Algas e seus Derivados, Lda., 3830-196 Ílhavo, Portugal; barbara.pitarma@algaplus.pt; madalena.mendes@algaplus.pt; helena.abreu@algaplus.pt
[5] Green Colab—Associação Oceano Verde, University of Algarve, Campus de Gambelas, 8005-139 Faro, Portugal;
[6] Amadeite SAS, Pôle biotechnologique du Haut du Bois, 56580 Bréhan, France; PNyvallCollen@olmix.com

* Correspondence: ana.moreira@ua.pt

Supplementary Table S1. Glycolipids identified by LC‒MS and MS/MS of *Ulva* spp. samples (mass error < 5 ppm).[1]

Lipid species (C:N)	Theoretical *m/z*	Observed *m/z*	Error (ppm)	Fatty acyl chain(s)	Formula
		**MGMG identified as $[M + NH_4]^+$**			
MGMG (16:4)	502.3016	502.3015	-0.2170	(16:4)	C25H44NO9
MGMG (16:3)	504.3173	504.3186	2.6590	**	C25H46NO9
MGMG (16:2)	506.3329	506.3335	1.1672	**	C25H48NO9
MGMG (16:1)	508.3486	508.3485	-0.1161	(16:1)	C25H50NO9
MGMG (16:0)	510.3642	510.3643	0.1959	(16:0)	C25H52NO9
MGMG (18:4)	530.3329	530.3332	0.5487	(18:4)	C27H48NO9
MGMG (22:5)[#]	584.3799	584.3797	-0.2721	**	C31H54NO9
		**DGMG identified as $[M + NH_4]^+$**			
DGMG (14:0)[§]	644.3857	644.3871	2.1198	**	C29H58NO14
DGMG (16:4)	664.3544	664.3552	1.1530	**	C31H54NO14
DGMG (16:2)	668.3857	668.3868	1.5949	**	C31H58NO14
DGMG (16:1)	670.4014	670.4019	0.7697	(16:1)	C31H60NO14
DGMG (16:0)	672.4170	672.4176	0.8923	(16:0)	C31H62NO14
		**MGDG identified as $[M + NH_4]^+$**			
MGDG (32:8)	732.4687	732.4679	-1.0922	**	C41H66NO10
MGDG (32:5)	738.5156	738.5172	2.1340	**	C41H72NO10
MGDG (32:4)	740.5307	740.5306	-0.1350	(16:4/16:0)	C41H74NO10
MGDG (32:0)[#]	748.5939	748.5930	-1.1675	(16:0/16:0)	C41H82NO10
MGDG (34:8)	760.5000	760.4999	-0.0973	(18:4/16:4)	C43H70NO10
MGDG (34:3)	770.5782	770.5783	0.0986	(18:3/16:0)	C43H80NO10
MGDG (36:9)	786.5156	786.5187	3.9414	**	C45H72NO10
MGDG (36:8)	788.5313	788.5306	-0.8497	**	C45H74NO10
MGDG (36:7)	790.5469	790.5478	1.1385	**	C45H76NO10
		**DGDG identified as $[M + NH_4]^+$**			
DGDG (32:4)	902.5841	902.5852	1.2187	(16:4/16:0)	C47H84O15N
DGDG (32:1)	908.6310	908.6302	-0.8804	(16:1/16:0)	C47H90O15N
DGDG (32:0)	910.6467	910.6457	-1.0981	(16:0/16:0)	C47H92O15N
DGDG (34:8)	922.5528	922.5519	-0.9756	(18:4/16:4)	C49H80O15N
DGDG (34:6)	926.5841	926.5833	-0.8634	**	C49H84O15N
DGDG (34:5)	928.5997	928.6020	2.4768	(18:1/16:4)	C49H86O15N
DGDG (34:4)	930.6154	930.6180	2.7939	**	C49H88O15N
DGDG (34:3)	932.6310	932.6318	0.8578	(18:3/16:0)	C49H90O15N
DGDG (34:2)	934.6467	934.6455	-1.2839	**	C49H92O15N
DGDG (34:1)	936.6623	936.6626	0.3203	(18:1/16:0)	C49H94O15N
DGDG (36:7)	952.5997	952.5976	-2.2045	**	C51H86O15N
DGDG (36:4)	958.6467	958.6458	-0.9388	(18:1/18:3)	C51H92O15N

Supplementary Table S1. [Continued]

Lipid species (C:N)	Theoretical *m/z*	Observed *m/z*	Error (ppm)	Fatty acyl chain(s)	Formula
**SQMG identified as [M - H]⁻**					
SQMG (14:0)§	527.2526	527.2532	1.1152	**	C23H43O11S
SQMG (16:1)§	553.2683	553.2690	1.3339	**	C25H45O11S
SQMG (16:0)	555.2839	555.2844	0.8788	(16:0)	C25H47O11S
SQMG (18:3)§	577.2683	577.2682	-0.1074	**	C27H45O11S
SQMG (18:1)	581.2996	581.3004	1.4433	**	C27H49O11S
**SQDG identified as [M - H]⁻**					
SQDG (28:0)§	737.4510	737.4516	0.8448	(14:0/14:0) and (12:0/16:0)	C37H69O12S
SQDG (30:1)§	763.4666	763.4666	-0.0354	(14:0/16:1)	C39H71O12S
SQDG (30:0)	765.4823	765.4831	1.0751	(14:0/16:0)	C39H73O12S
SQDG (32:4)	785.4510	785.4529	2.4483	(16:4/16:0)	C41H69O12S
SQDG (32:2)	789.4823	789.4831	1.0425	**	C41H73O12S
SQDG (32:1)	791.4979	791.4991	1.4820	(16:1/16:0) and (18:1/14:0)	C41H75O12S
SQDG (32:0)	793.5136	793.5140	0.5343	(16:0/16:0)	C41H77O12S
SQDG (34:7)§	807.4353	807.4347	-0.7765	**	C43H67O12S
SQDG (34:5)§	811.4666	811.4643	-2.8676	**	C43H71O12S
SQDG (34:4)	813.4823	813.4827	0.5200	(18:4/16:0)	C43H73O12S
SQDG (34:3)	815.4979	815.4987	0.9479	(18:3/16:0)	C43H75O12S
SQDG (34:1)	819.5292	819.5296	0.4551	(18:1/16:0)	C43H79O12S
SQDG (36:5)	839.4979	839.4969	-1.2234	**	C45H75O12S
SQDG (36:4)	841.5136	841.5139	0.3850	**	C45H77O12S
SQDG (36:2)	845.5449	845.5427	-2.5747	(20:2/16:0)	C45H81O12S
SQDG (36:1)	847.5605	847.5596	-1.0937	**	C45H83O12S
SQDG (38:0)	877.6075	877.6076	0.1402	**	C47H89O12S

[1]Observed *m/z* and respective error were checked for all samples, but those presented were obtained from PT sample (replicate 1), or if absent in this sample, from FR sample (replicate 1). Numbers in parenthesis (C:N) indicate the number of carbon atoms (C) and double bonds (N) in the fatty acyl chains. **, Lipid species identified only by retention time and mass accuracy. #, Not detected in PT samples. §, Not detected in FR samples.

Supplementary Table S2. Phospholipids identified by LC–MS and MS/MS of *Ulva* spp. samples (mass error < 5 ppm).[1]

Lipid species (C:N)	Theoretical *m/z*	Observed *m/z*	Error (ppm)	Fatty acyl chain(s)	Formula
**PC identified as [M + H]⁺** (determination of fatty acyl chains by MS/MS of [M + CH_3COO]⁻)					
PC (30:0)	706.5387	706.5384	-0.3991	*	C38H77NO8P
PC (32:2)	730.5387	730.5383	-0.5229	(16:1/16:1)	C40H77NO8P
PC (32:1)	732.5543	732.5530	-1.8169	(16:0/16:1)	C40H79NO8P
PC (34:3)	756.5543	756.5526	-2.2880	**	C42H79NO8P
PC (34:2)	758.5700	758.5699	-0.1081	(16:1/18:1)	C42H81NO8P
PC (34:1)	760.5856	760.5849	-0.9624	(16:0/18:1)	C42H83NO8P
PC (36:5)	780.5543	780.5531	-1.5771	*	C44H79NO8P
PC (36:4)	782.5700	782.5672	-3.5550	**	C44H81NO8P
PC (36:3)	784.5856	784.5843	-1.6977	(18:1/18:2)	C44H83NO8P

Supplementary Table S2. [Continued]

Lipid species (C:N)	Theoretical *m/z*	Observed *m/z*	Error (ppm)	Fatty acyl chain(s)	Formula
PC (36:2)	786.6013	786.6015	0.2771	(18:1/18:1)	C44H85NO8P
PC (38:6)	806.5700	806.5689	-1.3415	*	C46H81NO8P
PC (38:5)	808.5856	808.5837	-2.3894	**	C46H83NO8P
**LPC identified as [M + H]$^+$** (determination of fatty acyl chains by MS/MS of [M + CH_3COO]$^-$)					
LPC (14:0)	468.3090	468.3087	-0.6769	(14:0)	C22H47NO7P
LPC (16:1)	494.3247	494.3245	-0.3378	(16:1)	C24H49NO7P
LPC (16:0)	496.3403	496.3404	0.1692	(16:0)	C24H51NO7P
LPC (18:4)	516.3090	516.3071	-3.7129	**	C26H47NO7P
LPC (18:3)	518.3247	518.3222	-4.7596	**	C26H49NO7P
LPC (18:1)	522.3560	522.3560	0.0632	(18:1)	C26H53NO7P
LPC (20:5)	542.3247	542.3237	-1.7831	(20:5)	C28H49NO7P
LPC (20:4)	544.3403	544.3390	-2.4176	*	C28H51NO7P
LPC (22:6)	568.3403	568.3407	0.6757	**	C30H51NO7P
**PE identified as identified as [M - H]$^-$** (confirmation of PE polar head by MS/MS of [M + H]$^+$)					
PE (30:1)	660.4604	660.4609	0.7086	(14:0/16:1)	C35H67NO8P
PE (32:2)	686.4761	686.4768	1.0459	(16:1/16:1)	C37H69O8NP
PE (32:1)	688.4917	688.4922	0.6797	(16:0/16:1)	C37H71NO8P
PE (34:2)	714.5074	714.5077	0.4451	(16:1/18:2)	C39H73NO8P
PE (34:1)	716.5230	716.5219	-1.5799	**	C39H75NO8P
PE (36:2)	742.5387	742.5406	2.5830	**	C41H77NO8P
**LPE identified as [M - H]$^-$** (confirmation of PE polar head by MS/MS of [M + H]$^+$)					
LPE (16:1)	450.2621	450.2629	1.8500	(16:1)	C21H41NO7P
LPE (16:0)	452.2777	452.2783	1.2890	(16:0)	C21H43NO7P
LPE (18:2)[§]	476.2777	476.2782	1.0141	**	C23H43NO7P
LPE (18:1)	478.2934	478.2938	0.9053	**	C23H45NO7P
LPE (20:4)	500.2777	500.2782	0.9655	**	C25H43NO7P
LPE (22:5)	526.2934	526.2935	0.2527	*	C27H45NO7P
**PI identified as [M - H]$^-$**					
PI (34:2)	833.5180	831.4999	-3.0066	(16:0/18:2)	C43H78O13P
PI (34:3)	831.5024	833.5140	-4.7989	(16:0/18:3)	C43H76O13P
PI (34:1)	835.5337	835.5298	-4.6677	(16:0/18:1)	C43H80O13P
**LPI identified as [M - H]$^-$**					
LPI (16:0)	571.2883	571.2896	2.2020	(16:0)	C25H48O12P
**PG identified as [M - H]$^-$**					
PG (30:1)	691.4550	691.4562	1.7355	(14:0/16:1)	C36H68O10P
PG (32:2)	717.4707	717.4713	0.8363	(16:1/16:1)	C38H70O10P
PG (32:1)	719.4863	719.4866	0.4170	(16:0/16:1) and (14:0/18:1)	C38H72O10P
PG (34:5)	739.4550	739.4558	1.0819	(16:1/18:4) and (16:2/18:3)	C40H68O10P
PG (34:4)	741.4707	741.4711	0.5395	(16:1/18:3)	C40H70O10P
PG (34:3)	743.4863	743.4847	-2.1520	(16:1/18:2) and (16:0/18:3)	C40H72O10P

Supplementary Table S2. [Continued]

Lipid species (C:N)	Theoretical *m/z*	Observed *m/z*	Error (ppm)	Fatty acyl chain(s)	Formula
PG (34:2)	745.5020	745.5023	0.4024	(16:1/18:1) and (16:0/18:2)	C40H74O10P
PG (36:3)	771.5176	771.5182	0.7777	(18:1/18:2)	C42H76O10P
PG (36:2)	773.5333	773.5335	0.2586	(18:1/18:1)	C42H78O10P
**LPG identified as [M - H]$^-$**					
LPG (16:1)	481.2566	481.2572	1.1470	(16:1)	C22H42O9P
LPG (16:0)	483.2723	483.2734	2.2803	(16:0)	C22H44O9P
LPG (18:3)	505.2566	505.2582	3.0717	**	C24H42O9P
LPG (18:1)	509.2879	509.2886	1.2802	**	C24H46O9P

[1]Observed *m/z* and respective error were checked for all samples, but those presented were obtained from PT sample (replicate 1), or if absent in this sample, from FR sample (replicate 1). Numbers in parenthesis (C:N) indicate the number of carbon atoms (C) and double bonds (N) in the fatty acyl chains. *, Lipid species identified by retention time, mass accuracy and MS/MS of [M + H]$^+$ (without MS/MS in negative ion mode to confirm fatty acyl chains). **, Lipid species identified only by retention time and mass accuracy.

Supplementary Table S3. Betaine lipids identified by LC−MS and MS/MS of *Ulva* spp. samples (mass error < 5 ppm).[1]

Lipid species (C:N)	Theoretical *m/z*	Observed *m/z*	Error (ppm)	Fatty acyl chain(s)	Formula
**DGTS identified as [M + H]$^+$**					
DGTS (28:0)	656.5465	656.5463	-0.3046	(12:0/16:0) and (14:0/14:0)	C38H74O7N
DGTS (30:4)	676.5152	676.5152	0.0000	**	C40H70O7N
DGTS (30:1)	682.5622	682.5621	-0.1465	(14:0/16:1) and (14:1/16:0)	C40H76O7N
DGTS (30:0)	684.5778	684.5780	0.2922	(14:0/16:0)	C40H78O7N
DGTS (32:4)	704.5465	704.5465	0.0000	(14:0/18:4) and (16:0/16:4)	C42H74O7N
DGTS (32:3)	706.5622	706.5617	-0.7077	**	C42H76O7N
DGTS (32:2)	708.5778	708.5781	0.4234	(16:1/16:1) and (16:0/16:2)	C42H78O7N
DGTS (32:1)	710.5935	710.5934	-0.1407	(16:0/16:1) and (14:0/18:1)	C42H80O7N
DGTS (32:0)	712.6091	712.6080	-1.5436	(16:0/16:0)	C42H82O7N
DGTS (34:8)	724.5152	724.5167	2.0303	(16:4/18:4)	C44H70O7N
DGTS (34:7)	726.5309	726.5290	-2.5863	**	C44H72O7N
DGTS (34:6)	728.5465	728.5461	-0.5490	(16:2/18:4), (16:4/18:2) and (16:1/18:3)	C44H74O7N
DGTS (34:5)	730.5622	730.5625	0.4106	(16:4/18:1)	C44H76O7N
DGTS (34:4)	732.5778	732.5776	-0.2730	(16:0/18:4) and (16:1/18:3)	C44H78O7N
DGTS (34:3)	734.5935	734.5927	-1.0890	(16:0/18:3)	C44H80O7N
DGTS (34:2)	736.6091	736.6085	-0.8145	(16:0/18:2) and (16:1/18:1)	C44H82O7N
DGTS (34:1)	738.6248	738.6244	-0.5415	(16:0/18:1) and (16:1/18:0)	C44H84O7N
DGTS (36:8)	752.5465	752.5458	-0.9568	(18:4/18:4)	C46H74O7N
DGTS (36:7)	754.5622	754.5600	-2.9156	(18:3/18:4) and (18:2/18:5)[◊]	C46H76O7N
DGTS (36:6)	756.5778	756.5761	-2.2470	**	C46H78O7N
DGTS (36:5)	758.5935	758.5935	0.0000	(18:1/18:4)	C46H80O7N

Supplementary Table S3. [Continued]

Lipid species (C:N)	Theoretical *m/z*	Observed *m/z*	Error (ppm)	Fatty acyl chain(s)	Formula
DGTS (36:4)	760.6091	760.6081	-1.3147	(18:0/18:4), (18:1/18:3) and (16:0/20:4)◊	C46H82O7N
DGTS (36:2)	764.6404	764.6404	0.0000	(18:1/18:1) and (16:0/20:2)	C46H86O7N
DGTS (38:9)	778.5622	778.5597	-3.2110	(20:5/18:4) and (16:4/22:5)	C48H76O7N
DGTS (38:8)	780.5778	780.5761	-2.1779	**	C48H78O7N
DGTS (38:7)	782.5935	782.5905	-3.8334	(20:4/18:3)	C48H80O7N
DGTS (38:6)	784.6091	784.6071	-2.5490	(16:1/22:5) and (20:4/18:2)	C48H82O7N
DGTS (38:5)	786.6248	786.6249	0.1271	(16:0/22:5) and (20:4/18:1)	C48H84O7N
DGTS (38:0)	796.7030	796.7014	-2.0083	(16:0/22:0)	C48H94O7N
DGTS (40:9)	806.5935	806.5944	1.1158	(22:5/18:4)	C50H80O7N
DGTS (40:8)	808.6091	808.6081	-1.2367	**	C50H82O7N
DGTS (40:7)	810.6248	810.6229	-2.3439	**	C50H84O7N
DGTS (40:6)	812.6404	812.6410	0.7383	**	C50H86O7N
DGTS (40:4)	816.6717	816.6736	2.3265	(22:0/18:4)	C50H90O7N
DGTS (42:10)	832.6091	832.6093	0.2066	(22:5/20:5)	C52H82O7N
DGTS (44:10)	860.6404	860.6398	-0.7309	(22:5/22:5)	C54H86O7N
**MGTS identified as [M + H]$^+$**					
MGTS (14:0)	446.3482	446.3479	-0.5915	(14:0)	C24H48O6N
MGTS (16:4)	466.3169	466.3169	0.0772	(16:4)	C26H44O6N
MGTS (16:2)	470.3482	470.3483	0.2891	(16:2)	C26H48O6N
MGTS (16:1)	472.3638	472.3639	0.1821	(16:1)	C26H50O6N
MGTS (16:0)	474.3795	474.3792	-0.6324	(16:0)	C26H52O6N
MGTS (18:5)	492.3325	492.3307	-3.6845	(18:5)◊	C28H46O6N
MGTS (18:4)	494.3482	494.3474	-1.5455	(18:4)	C28H48O6N
MGTS (18:3)	496.3638	496.3628	-2.0429	(18:3)◊	C28H50O6N
MGTS (18:1)	500.3951	500.3947	-0.8273	(18:1)	C28H54O6N
MGTS (20:5)	520.3638	520.3633	-0.9878	(20:5)	C30H50O6N
MGTS (20:4)	522.3795	522.3779	-2.9940	(20:4)◊	C30H52O6N
MGTS (20:1)	528.4264	528.4261	-0.5942	(20:1)	C30H58O6N
MGTS (20:0)	530.4421	530.4421	0.0679	(20:0)	C30H60O6N
MGTS (22:5)	548.3951	548.3952	0.1568	(22:5)	C32H54O6N
MGTS (22:0)	558.4734	558.4733	-0.1146	(22:0)	C32H64O6N

[1] Observed *m/z* and respective error were checked for all samples, but those presented were obtained from PT sample (replicate 1), or if absent in this sample, from FR sample (replicate 1). Numbers in parenthesis (C:N) indicate the number of carbon atoms (C) and double bonds (N) in the fatty acyl chains. ◊, with contribution of sodium adducts. **, Lipid species identified only by retention time and mass accuracy.

Supplementary Table S4. Literature review data on the proximate composition and fatty acid profile (obtained by analysis of fatty acid methyl ester derivatives) found for *Ulva* genus in this study and other published studies.

Reference	Sample	Wild or cultivation	Location	Harvest time	Proximate composition (%DW)[a]				Most abundant FA (% of total FA)[e]
					Ash	Proteins[b]	Sugars[c]	Lipids[d]	
This study	*Ulva rigida*	Cultivation	Ria de Aveiro, Portugal	May 2017	32.5	11.1	55.2	1.1	16:0 (42), 18:1 (21), 18:3*n*-3 (5), 16:1*n*-7 (5), 18:0 (4), 18:4*n*-3 (4)
	Ulva spp.	Wild	Brittany, France		13.6	15.6	68.6	2.2	16:0 (36), 18:4*n*-3 (14), 16:4*n*-3 (10), 18:1 (9), 18:0 (6), 18:3*n*-3 (6)
Fleurence et al. [1]	*Ulva rotundata*	Wild	Brittany, France	Dec 1991				1.9	16:0 (34), 18:4*n*-3 (14), 18:1*n*-7 (14), 16:4*n*-3 (10), 18:3*n*-3 (10), 16:1*n*-7 (4)
Nelson et al. [2]	*Ulva lobata*	Wild	San Diego, California	Dec 1997				2.5	16:0 (22), 18:3*n*-3 (21), C16 PUFA (20), 18:4*n*-3 (12), 18:1*n*-7 (8), 18:2*n*-6 (4)
		Wild	San Diego, California	March 1998				2.9	18:3*n*-3 (25), 16:0 (21), C16 PUFA (20), 18:4*n*-3 (14), 18:1*n*-7 (7), 16:1*n*-13 (4)
		Wild	San Diego, California	July 1998				2.0	16:0 (28), 18:3*n*-3 (17), C16 PUFA (15), 18:2*n*-6 (11), 18:1*n*-7 (9), 18:4*n*-3 (7)
		Wild	San Diego, California	Oct 1998				2.1	16:0 (25), 18:3*n*-3 (24), C16 PUFA (17), 18:4*n*-3 (12), 18:1*n*-7 (9), 16:1*n*-13 (3)
Kumari et al. [3]	*Ulva tubulosa*	Wild	Coast of Gujarat, India	Jan-April 2008				2.1	16:0 (49), 18:1*n*-9 (19), 18:2*n*-6 (11), 22:6*n*-3 (5), 18:0 (4), 20:5*n*-3 (2)
	Ulva linza	Wild	Coast of Gujarat, India	Jan-April 2008				2.1	16:0 (41), 18:1*n*-9 (15), 18:2*n*-6 (11), 22:6*n*-3 (6), 20:5*n*-3 (4), 18:0 (4)
	Ulva fasciata	Wild	Coast of Gujarat, India	Jan-April 2008				1.8	16:0 (53), 18:1*n*-9 (12), 18:2*n*-6 (12), 22:6*n*-3 (6), 18:0 (4), 20:5*n*-3 (2)
	Ulva rigida	Wild	Coast of Gujarat, India	Jan-April 2008				2.0	16:0 (48), 18:1*n*-9 (19), 18:2*n*-6 (9), 22:6*n*-3 (6), 18:0 (4), 16:1*n*-7 (4)
	Ulva reticulata	Wild	Coast of Gujarat, India	Jan-April 2008				2.0	16:0 (52), 18:1*n*-9 (17), 18:2*n*-6 (8), 18:0 (6), 16:1*n*-7 (4), 22:6*n*-3 (3)
	Ulva lactuca	Wild	Coast of Gujarat, India	Jan-April 2008				1.3	16:0 (43), 18:1*n*-9 (18), 18:2*n*-6 (9), 16:1*n*-7 (6), 18:0 (3), 18:3*n*-6 (3)
	Ulva sp.	Wild	Coast of Gujarat, India	Jan-April 2008				1.8	16:0 (50), 18:1*n*-9 (16), 18:2*n*-6 (10), 16:1*n*-7 (4), 14:1 (4), 22:6*n*-3 (3)
van Ginneken et al. [4]	*Ulva lactuca*	Wild	Eastern Scheldt, the Netherlands	Sep-Oct 2009				2.2	18:2 (25), 18:3 (20), 18:1*n*-9 (20), 16:0 (12), 18:4*n*-3 (8), 18:1 (4)
Yaich et al. [5]	*Ulva lactuca*	Wild	Monastir, Tunisia	July 2007	19.6	8.5	54.9	7.9	16:0 (59), 18:1*n*-9 (16), 16:1*n*-9 (7), 22:0 (4), 18:3*n*-3 (3), 18:2*n*-6 (2)

Supplementary Table S4. [Continued]

Reference	Sample	Wild or cultivation	Location	Harvest time	Proximate composition (%DW)[a]				Most abundant FA (% of total FA)[e]
					Ash	Proteins[b]	Sugars[c]	Lipids[d]	
Gosch et al. [6]	*Ulva fleximosa*	Wild	North Queensland, Australia	na				6.5	16:0 (25), 18:2*n*-6 (13), 18:1*n*-9 (10), 18:3*n*-3 (7),14:0 (5), 16:4*n*-3 (5)
	Ulva rigida	Wild	North Queensland, Australia	na				2.5, 3.2	16:0 (29), 18:4*n*-3 (12), 18:3*n*-3 (11), 16:4*n*-3 (9), 18:1*n*-9 (8), 16:1*n*-7 (5)
Khairy et al. [7]	*Ulva lactuca*	Wild	Abu Qir Bay, Egypt	April 2010	22.1	20.1	44.8	4.1	16:0 (60), 22:6*n*-3 (10), 13:0 (8), 15:1 (5), 14:1(5), 20:0 (2)
	Ulva lactuca	Wild	Abu Qir Bay, Egypt	Aug 2010	17.6	17.9	46.4	3.6	16:0 (52), 22:6*n*-3(13), 13:0 (6), 18:0 (5), 15:1 (5)
	Ulva lactuca	Wild	Abu Qir Bay, Egypt	Oct 2010	23.2	16.8	42.1	3.1	16:0 (53), 18:1 (12), 22:6*n*-3 (5), 18:0 (5), 13:0 (4), 22:1 (3)
Pereira et al. [8]	*Ulva* sp.	Wild	Algarve, Portugal	May 2010					16:0 (50), 18:3*n*-3 (16), 16:1*n*-7 (11), 18:2*n*-6 (6), 18:1*n*-9 (6)
Maehre et al. [9]	*Ulva lactuca*	Wild	Norway	June 2012	29.3	8.7		2.6	16:0 (26), 18:3*n*-3 (15), 18:1*n*-7 (15), 18:2*n*-6 (11), 16:4*n*-3 (7), 18:4*n*-3 (6)
Kendel et al. [10]	*Ulva armoricana*	Wild	Brittany, France	June 2012				2.6	16:0 (42), 18:1*n*-7 (17), 18:4*n*-3 (9), 18:2*n*-3 (8), 16:4*n*-3 (6), 18:2*n*-6 (4)
Serviere-Zaragoza et al. [11]	*Ulva lactuca*	Wild	El Rincón, Mexico	May 2003					16:0 (35), 18:4*n*-3 (19), 18:3*n*-3 (16), 18:1*n*-7 (12), 16:4*n*-3 (9), 18:2*n*-6 (4)
	Ulva lactuca	Wild	El Rincón, Mexico	Aug 2003					16:0 (34), 18:4*n*-3 (15), 18:1*n*-7 (14), 18:3*n*-3 (12), 16:4*n*-3 (8),16:1*n*-7 (5)
	Ulva lactuca	Wild	El Rincón, Mexico	Nov 2003					16:0 (32), 18:4*n*-3 (17), 18:1*n*-7 (15), 18:3*n*-3 (13), 16:4*n*-3 (10), 16:1*n*-7 (5)
	Ulva lactuca	Wild	El Rincón, Mexico	Feb 2004					16:0 (32), 18:4*n*-3 (18), 18:1*n*-7 (16), 18:3*n*-3 (15), 16:4*n*-3 (7), 16:1*n*-7 (4)
	Ulva lactuca	Wild	El Rincón, Mexico	May 2004					16:0 (37), 18:1*n*-7 (18), 18:4*n*-3 (16), 18:3*n*-3 (14), 16:1*n*-7 (5), 16:4*n*-3 (4)
Paiva et al. [12]	*Ulva compressa*	Wild	Azores, Portugal	April 2013				1.7	16:0 (31), 18:1*n*-11 (19), 18:3*n*-6 (17), 17:1*n*-7 (8), 18:2*n*-6 (7), 18:1*n*-9 (3)
	Ulva rigida	Wild	Azores, Portugal	April 2013				1.0	16:0 (43), 18:3*n*-6 (17), 18:1*n*-9 (16), 18:2*n*-6 (5), 20:4*n*-6 (3), 20:1*n*-9 (3)

Supplementary Table S4. [Continued]

Reference	Sample	Wild or cultivation	Location	Harvest time	Proximate composition (%DW)[a]				Most abundant FA (% of total FA)[e]
					Ash	Proteins[b]	Sugars[c]	Lipids[d]	
Cardoso et al. [13]	*Ulva lactuca*	Cultivation	Olhão, Portugal	July[†]					16:0 (19), 16:3*n*-3 + 16:4*n*-3 (11), 18:2*n*-6 (10), 14:0 (9), 18:1*n*-7 + 18:1*n*-9 (8), 20:4*n*-6 (2)
	Ulva prolifera	Cultivation	Olhão, Portugal	July[†]					18:2*n*-6 (22), 16:0 (21), 18:1*n*-7 + 18:1*n*-9 (12), 14:0 (11), 16:3*n*-3 + 16:4*n*-3 (9), 20:5*n*-3 (2)
	Ulva intestinalis	Cultivation	Olhão, Portugal	July[†]					16:0 (19), 16:3*n*-3 + 16:4*n*-3 (11), 14:0 (9), 18:2*n*-6 (8), 18:1*n*-7+18:1*n*-9 (7), 20:4*n*-6 (2)
Gadberry et al. [14]	*Ulva* spp.	Cultivation	Manchester, USA	Aug 2013-Sep2014	32	29.5		3.0	
Neto et al. [15]	*Ulva rigida*	Cultivation	Ria de Aveiro, Portugal	July 2016	31.7	9.3	58.1	0.9	
Lopes et al. [16]	*Ulva rigida*	Cultivation	Ria de Aveiro, Portugal	Nov 2016	26.47	17.75	53.25	2.53	16:0 (43), 18:0 (19), 18:4*n*-3 (9), 18:1 (9), 18:3*n*-3 (4), 16:4*n*-3 (4)
Mohy El-Din et al. [17]	*Ulva lactuca*	Wild	Abu Qir Bay, Egypt	Summer 2016	24.5	20.3	19.5	3.8	
	Ulva lactuca	Wild	Abu Qir Bay, Egypt	Autumn 2016	21.5	18.2	18.4	3.2	
	Ulva lactuca	Wild	Abu Qir Bay, Egypt	Winter 2017	28.9	15.7	17.2	2.8	
	Ulva lactuca	Wild	Abu Qir Bay, Egypt	Spring 2017	28.3	23.2	18.9	4.1	
da Costa et al. [18]	*Ulva rigida*	Cultivation	Ria de Aveiro, Portugal	Summer 2018	27.79	8.55	63.26	0.4	
	Ulva spp.	Wild	Ria de Arousa, Spain	Summer 2018	25.4	6.6	67.5	0.5	
	Ulva spp.	Wild	Ria de Pontevedra, Spain	Summer 2018	22.2	9.9	66.2	1.8	
	Ulva spp.	Wild	Ria de Vigo, Spain	Summer 2018	25.6	4.7	69.2	0.5	
	Ulva spp.	Wild	Viana do Castelo, Portugal	Summer 2018	20.2	8.6	70.6	0.6	
	Ulva spp.	Wild	Peniche, Portugal	Summer 2018	27.3	18.1	53.4	1.2	
	Ulva spp.	Wild	Sado Estuary, Portugal	Summer 2018	13.5	12.6	73.6	0.4	

Supplementary Table S4. [Continued]

Reference	Sample	Wild or cultivation	Location	Harvest time	Proximate composition (%DW)[a]				Most abundant FA (% of total FA)[e]
					Ash	Proteins[b]	Sugars[c]	Lipids[d]	
da Costa et al. [18]	*Ulva* spp.	Wild	Albufeira, Portugal	Summer 2018	18.5	9.1	71.9	0.5	
	Ulva spp.	Wild	Ria Formosa, Portugal	Summer 2018	14.77	6.2	78.7	0.3	
Moreira et al. [19]	*Ulva rigida*	Cultivation	Ria de Aveiro, Portugal	August 2016				1.7	16:0 (44), 18:0 (13), 18:1 (11), 18:3*n*-3 (8), 18:4*n*-3 (8), 16:4*n*-3 (6)
	Ulva rigida	Cultivation	Ria de Aveiro, Portugal	November 2016				2.7	16:0 (31), 18:4*n*-3 (14), 18:0 (12), 16:4*n*-3 (11), 18:1 (8), 18:3*n*-3 (8)
	Ulva rigida	Cultivation	Ria de Aveiro, Portugal	March 2017				3.4	16:0 (27), 18:4*n*-3 (20), 16:4*n*-3 (14), 18:3*n*-3 (10), 18:0 (10), 18:1 (7)
	Ulva rigida	Cultivation	Ria de Aveiro, Portugal	May 2017				2.2	16:0 (30), 18:4*n*-3 (17), 16:4*n*-3 (12), 18:3*n*-3 (10), 18:1 (9), 18:0 (9)
Roleda et al. [20]	*Ulva lactuca*	Cultivation	Nordland, Norway	2016-2017	20-25‡	13.1-14.5‡	41.4-58.8‡	9.7-13.7‡	

[a] Values presented (round to decimals) are mean values from results expressed as percentage of the dry weight (%DW).

[b] Protein content determined using nitrogen-protein conversion factor 6.25, except in studies of Maehre et al and Rodela et al (sum of total amino acids). In studies using nitrogen-protein conversion, different methods were used to determine nitrogen content, namely elemental analysis and Kjeldahl method.

[c] Carbohydrates (and others) determined by difference, except in studies of Roleda et al. (sum of neutral and acid monosaccharides), and Khairy et al. and Mohy El-Din et al. (Dubois method). Value from the study of Yaich et al. is related to total dietary fiber.

[d] Different adaptations of Bligh and Dyer and Folch methods or Soxhlet extraction were used to obtain lipid extracts.

[e] Values in parentheses (round to units) are mean values from results expressed as percentage of the total of fatty acids (FA).

† Year not available, ‡ Range of different strains, na - not available

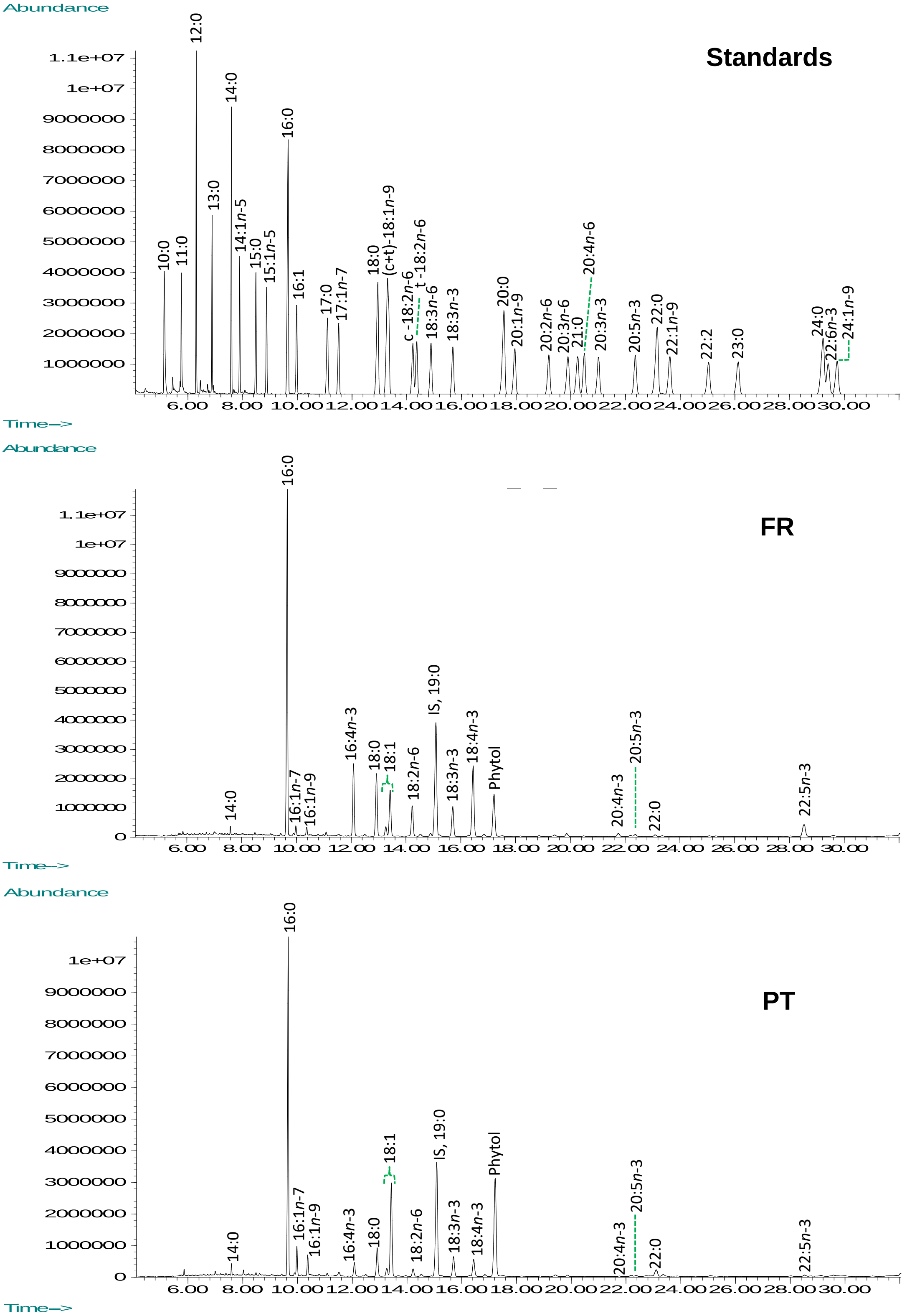

Supplementary Figure S1. Representative examples of total ion chromatograms (TIC) obtained by GC–MS analysis of the FAME standards (Supelco 37 Component FAME Mix, Sigma-Aldrich, Darmstadt, Germany) and the polar lipids extracts of *Ulva* spp. from Portugal (PT) and France (FR).

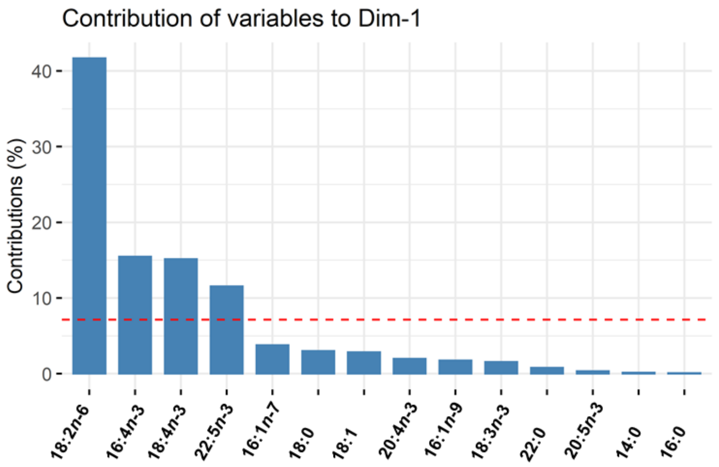

Supplementary Figure S2. Variables ordered by contribution (%) to dimension (dim) 1 of the principal component analysis (PCA) of esterified fatty acids dataset.

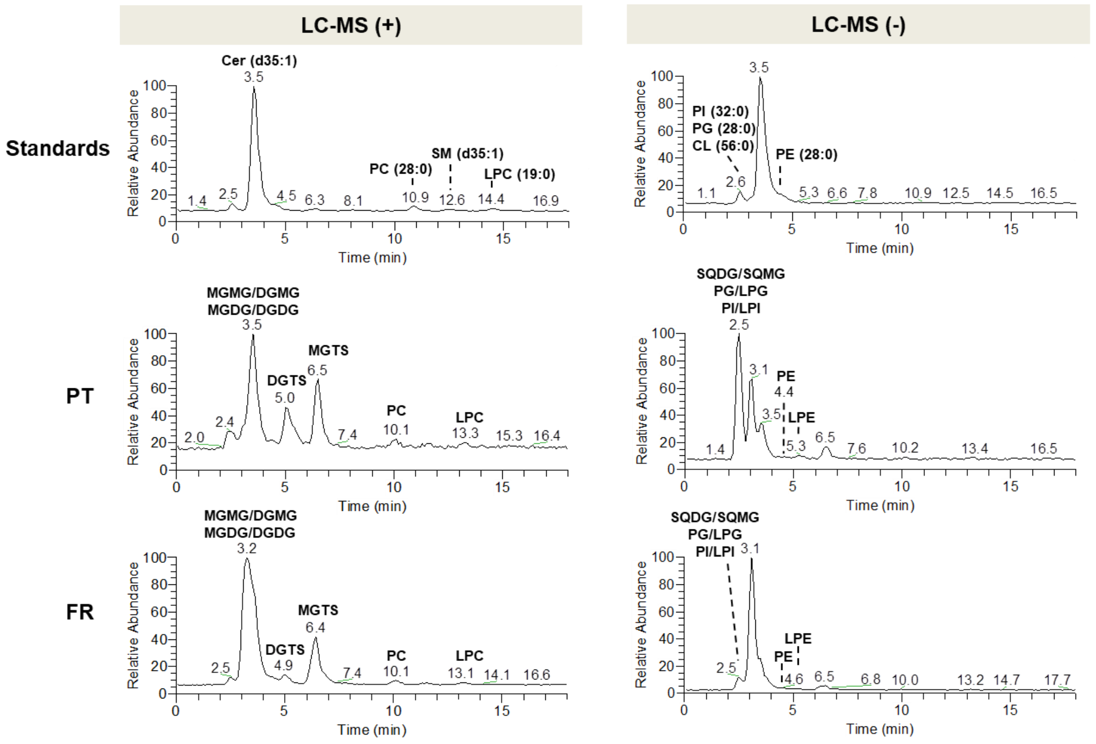

Supplementary Figure S3. Representative examples of total ion chromatograms (TIC) obtained by LC–MS analysis of the lipid standards and the polar lipids extracts of *Ulva* spp. from Portugal (PT) and France (FR), acquired on positive mode (+) and negative mode (-).

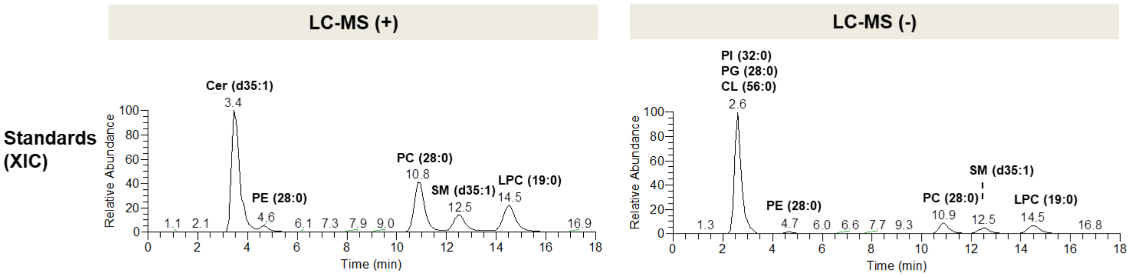

Supplementary Figure S4. Extracted ion chromatograms (XIC) obtained by LC–MS analysis of the lipid standards, acquired on positive mode (+) and negative mode (-).

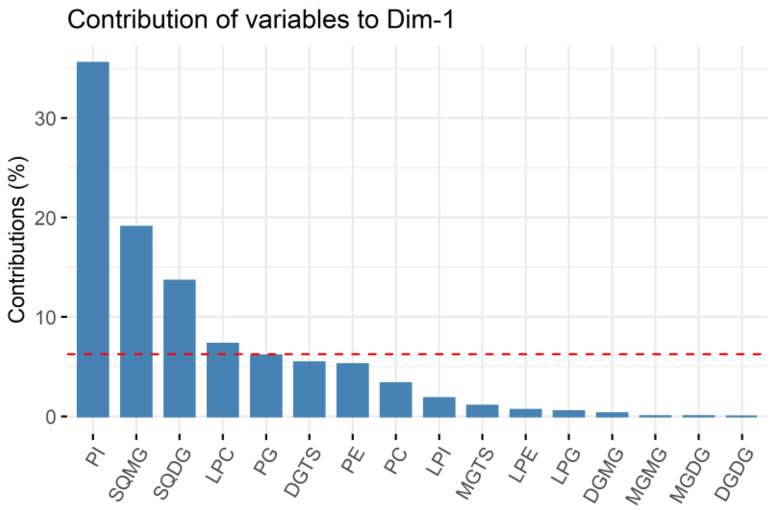

Supplementary Figure S5. Variables ordered by contribution (%) to dimension (dim) 1 of the principal component analysis (PCA) of lipid classes dataset.

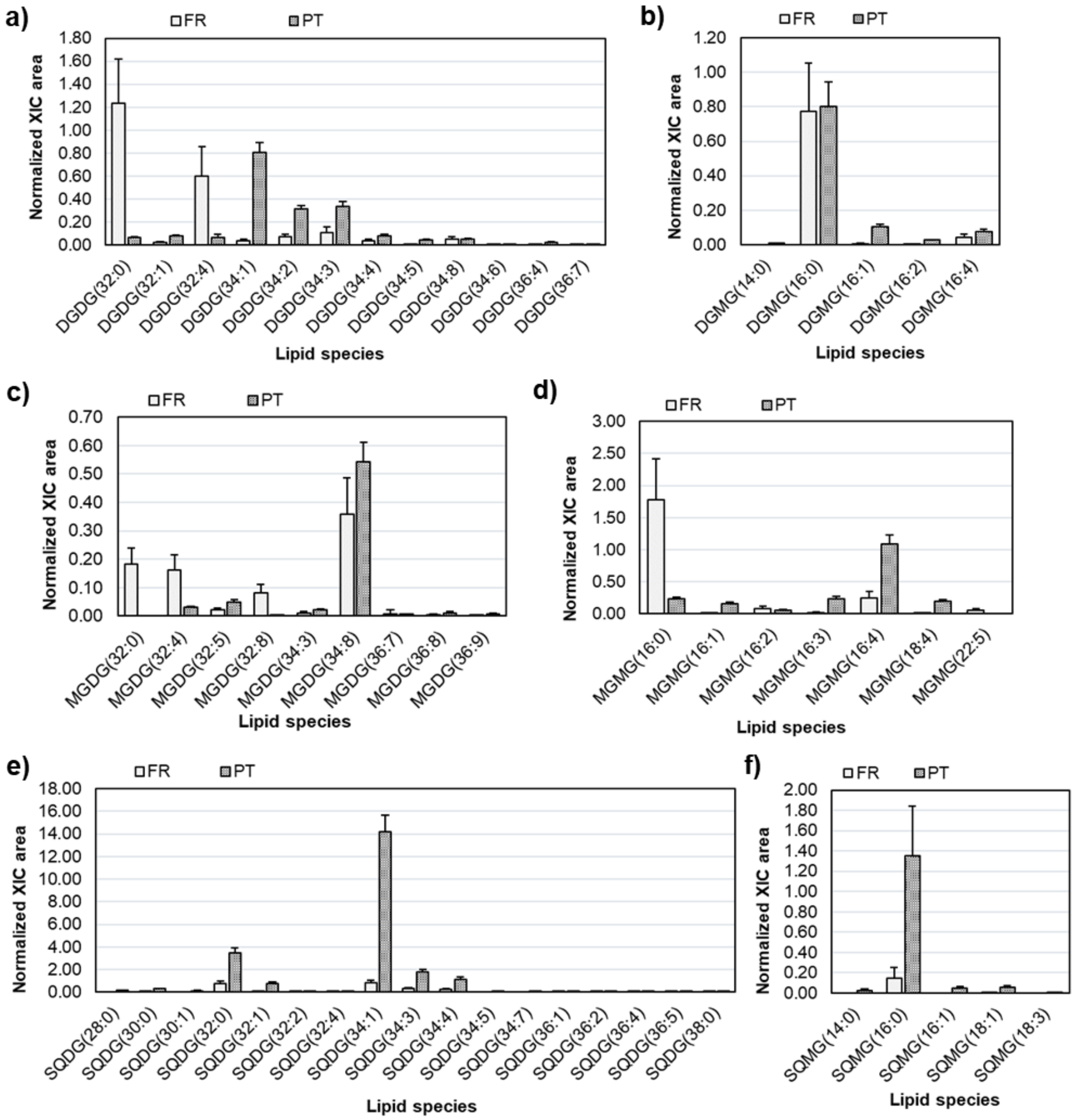

Supplementary Figure S6. Abundance (normalized XIC area) of glycolipid species identified by LC–MS and MS/MS of *Ulva* spp. samples from France (FR) and Portugal (PT), presented by class: a) DGDG, b) DGMG, c) MGDG, d) MGMG, e) SQDG, and f) SQMG species. Lipid species are labelled as follows: AAAA(C:D) (AAAA, lipid class abbreviation; C, number of carbon atoms in fatty acid(s); D, number of double bonds in fatty acid(s)).

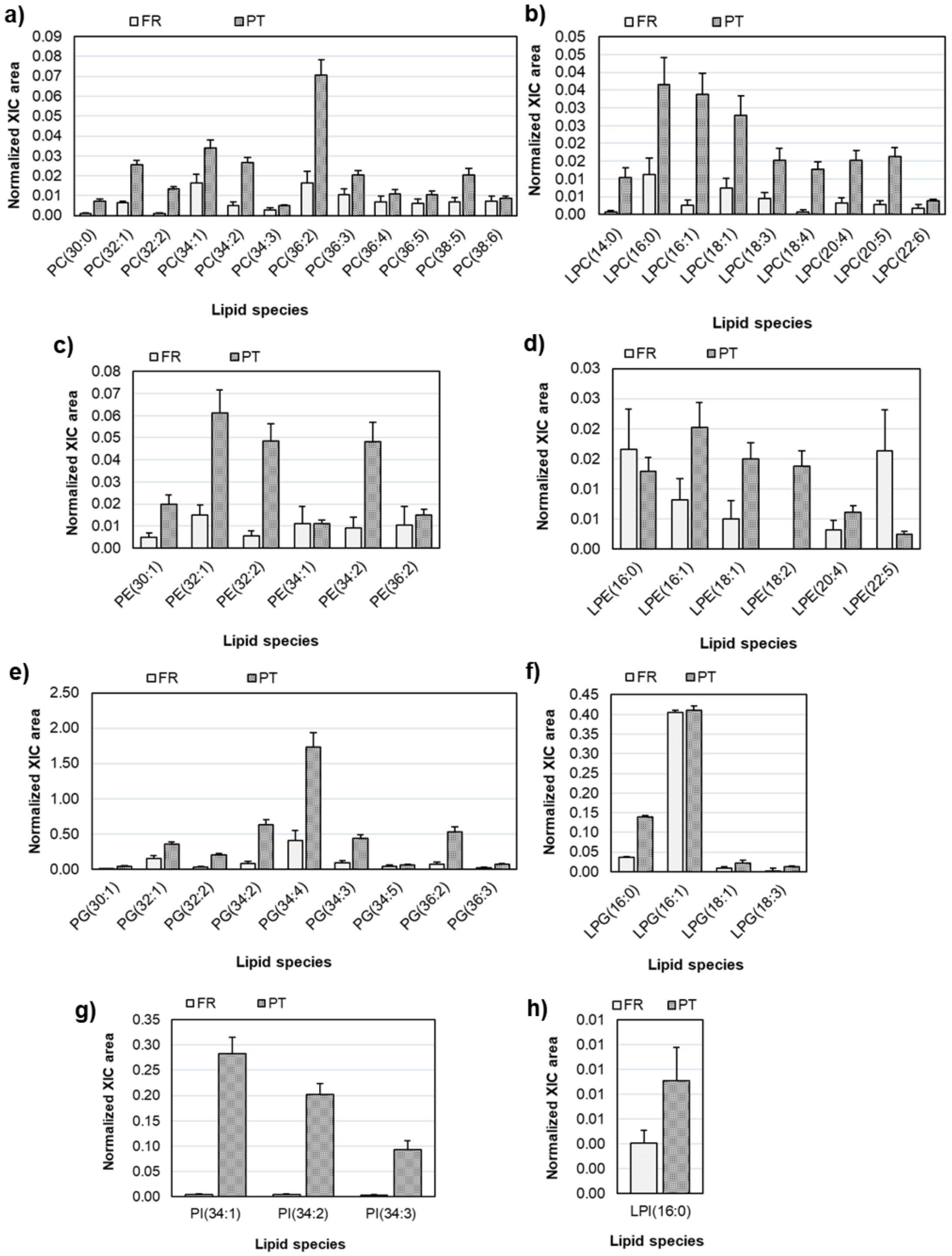

Supplementary Figure S7. Abundance (normalized XIC area) of phospholipid species identified by LC–MS and MS/MS of *Ulva* spp. samples from France (FR) and Portugal (PT), presented by class: a) PC, b) LPC, c) PE, d) LPE, e) PG, f) LPG, g) PI, and h) LPI species. Lipid species are labelled as follows: AAAA(C:D) (AAAA, lipid class abbreviation; C, number of carbon atoms in fatty acid(s); D, number of double bonds in fatty acid(s)).

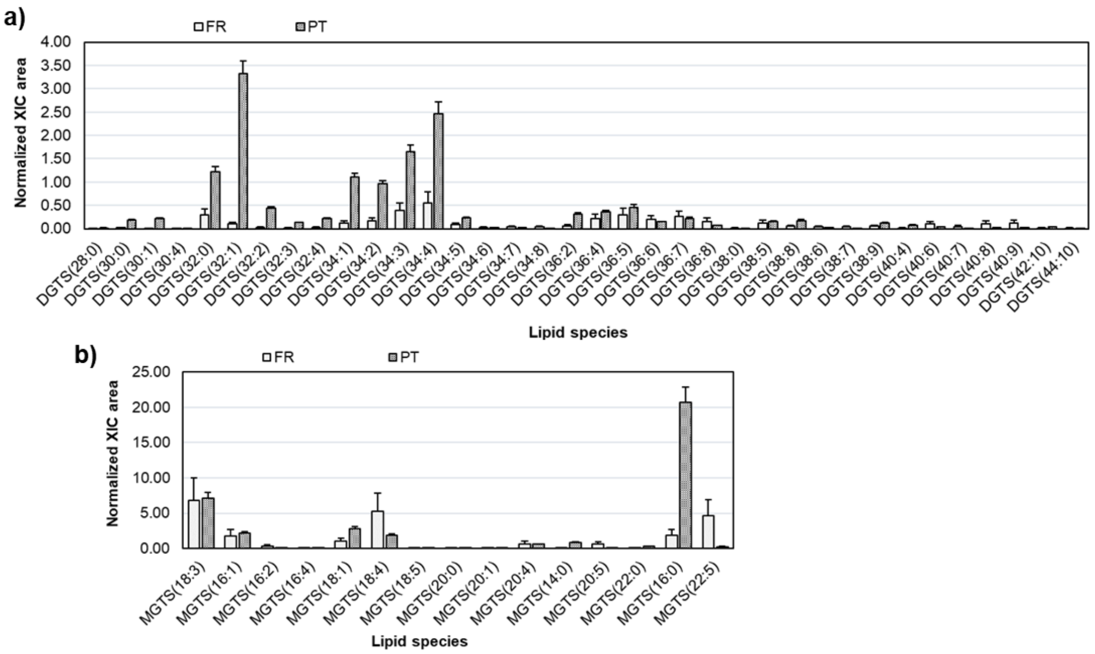

Supplementary Figure S8. Abundance (normalized XIC area) of betaine lipid species identified by LC–MS and MS/MS of *Ulva* spp. samples from France (FR) and Portugal (PT), presented by class: a) DGTS and b) MGTS species. Lipid species are labelled as follows: AAAA(C:D) (AAAA, lipid class abbreviation; C, number of carbon atoms in fatty acid(s); D, number of double bonds in fatty acid(s)).

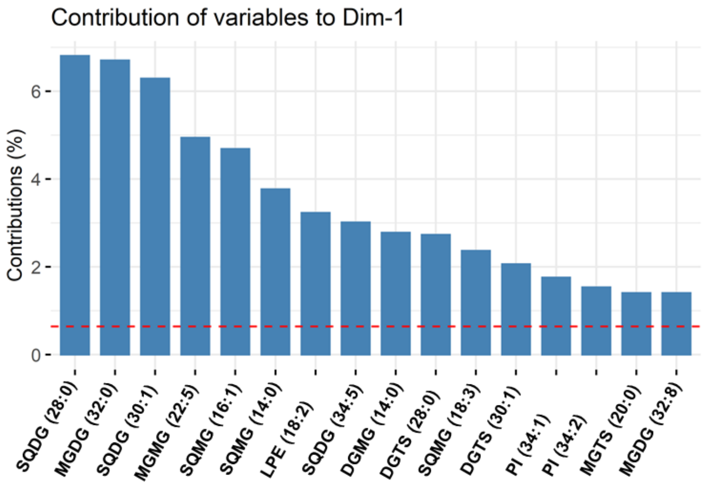

Supplementary Figure S9. Variables ordered by contribution (%) to dimension (dim) 1 of the principal component analysis (PCA) of lipid species dataset. Lipid species are labelled as follows: AAAA(C:D) (AAAA, lipid class abbreviation; C, number of carbon atoms in fatty acid(s); D, number of double bonds in fatty acid(s)).

REFERENCES

1. Fleurence, J.; Gutbier, G.; Mabeau, S.; Leray, C. Fatty acids from 11 marine macroalgae of the French Brittany coast. *J. Appl. Phycol.* **1994**, *6*, 527-532.

2. Nelson, M.M.; Phleger, C.F.; Nichols, P.D. Seasonal lipid composition in macroalgae of the northeastern pacific ocean. *Bot. Mar.* **2002**, *45,* 58-65.

3. Kumari, P.; Kumar, M.; Gupta, V.; Reddy, C.R.K.; Jha, B. Tropical marine macroalgae as potential sources of nutritionally important PUFAs. *Food Chem.* **2010**, *120*, 749-757.

4. van Ginneken, V.J.; Helsper, J.P.; de Visser, W.; van Keulen, H.; Brandenburg, W.A. Polyunsaturated fatty acids in various macroalgal species from North Atlantic and tropical seas. *Lipids Health Dis* **2011**, *10,* 10-104.

5. Yaich, H.; Garna, H.; Besbes, S.; Paquot, M.; Blecker, C.; Attia, H. Chemical composition and functional properties of *Ulva lactuca* seaweed collected in Tunisia. *Food Chem.* **2011**, *128,* 895-901.

6. Gosch, B.J.; Magnusson, M.; Paul, N.A.; de Nys, R. Total lipid and fatty acid composition of seaweeds for the selection of species for oil-based biofuel and bioproducts. *GCB Bioenergy* **2012**, *4,* 919-930.

7. Khairy, H.M.; El-Shafay, S.M. Seasonal variations in the biochemical composition of some common seaweed species from the coast of Abu Qir Bay, Alexandria, Egypt. *Oceanologia* **2013**, *55*, 435-452.

8. Pereira, H.; Barreira, L.; Figueiredo, F.; Custódio, L.; Vizetto-Duarte, C.; Polo, C.; Rešek, E.; Engelen, A.; Varela, J. Polyunsaturated fatty acids of marine macroalgae: potential for nutritional and pharmaceutical applications. *Mar. Drugs* **2012**, *10.*

9. Maehre, H.K.; Malde, M.K.; Eilertsen, K.E.; Elvevoll, E.O. Characterization of protein, lipid and mineral contents in common Norwegian seaweeds and evaluation of their potential as food and feed. *J Sci Food Agric* **2014**, *94*, 3281-3290.

10. Kendel, M.; Wielgosz-Collin, G.; Bertrand, S.; Roussakis, C.; Bourgougnon, N.; Bedoux, G. Lipid composition, fatty acids and sterols in the seaweeds *Ulva armoricana*, and *Solieria chordalis* from Brittany (France): An analysis from nutritional, chemotaxonomic, and antiproliferative activity perspectives. *Mar. Drugs* 2015, 13, 5606-5628.

11. Serviere-Zaragoza, E.; Hurtado, M.A.; Manzano-Sarabia, M.; Mazariegos-Villarreal, A.; Reza, M.; Arjona, O.; Palacios, E. Seasonal and interannual variation of fatty acids in macrophytes from the Pacific coast of Baja California Peninsula (Mexico). *J. Appl. Phycol.* **2015**, *27,* 1297-1306.

12. Paiva, L.; Lima, E.; Neto, A.I.; Marcone, M.; Baptista, J. Health-promoting ingredients from four selected Azorean macroalgae. *Food Res Int* **2016**, *89,* 432-438.

13. Cardoso, C.; Ripol, A.; Afonso, C.; Freire, M.; Varela, J.; Quental-Ferreira, H.; Pousão-Ferreira, P.; Bandarra, N. Fatty acid profiles of the main lipid classes of green seaweeds from fish pond aquaculture. *Food Sci Nutr* **2017**, *5,* 1186-1194.

14. Gadberry, B.A.; Colt, J.; Maynard, D.; Boratyn, D.C.; Webb, K.; Johnson, R.B.; Saunders, G.W.; Boyer, R.H. Intensive land-based production of red and green macroalgae for human consumption in the Pacific Northwest: an evaluation of seasonal growth, yield, nutritional composition, and contaminant levels. *Algae* **2018**, *33,* 109-125.

15. Neto, R.T.; Marçal, C.; Queirós, A.S.; Abreu, H.; Silva, A.M.S.; Cardoso, S.M. Screening of *Ulva rigida*, *Gracilaria* sp., *Fucus vesiculosus* and *Saccharina latissima* as functional ingredients. *Int. J. Mol. Sci.* **2018**, *19*, 2987.

16. Lopes, D.; Moreira, A.S.P.; Rey, F.; da Costa, E.; Melo, T.; Maciel, E.; Rego, A.; Abreu, M.H.; Domingues, P.; Calado, R.; Lillebø, A.I.; Domingues, M.R. Lipidomic signature of the green macroalgae *Ulva rigida* farmed in a sustainable integrated multi-trophic aquaculture. *J. Appl. Phycol.* **2019**, *31*, 1369–1381.

17. Mohy El-Din, S.M. Temporal variation in chemical composition of *Ulva lactuca* and *Corallina mediterranea*. *Int. J. Environ. Sci. Technol.* **2019**, *16*, 5783-5796.

18. da Costa, E.; Ricardo, F.; Melo, T.; Mamede, R.; Abreu, M.H.; Domingues, P.; Domingues, M.R.; Calado, R. Site-specific lipidomic signatures of sea lettuce (*Ulva* spp., Chlorophyta) hold the potential to trace their geographic origin. *Biomolecules* **2020**, *10*, 489.

19. Moreira, A.S.P.; da Costa, E.; Melo, T.; Sulpice, R.; Cardoso, S.M.; Pitarma, B.; Pereira, R.; Abreu, M.H.; Domingues, P.; Calado, R.; Domingues, M.R. Seasonal plasticity of the polar lipidome of *Ulva rigida* cultivated in a sustainable integrated multi-trophic aquaculture. *Algal Res.* **2020**, *49*, 101958.

20. Roleda, M.Y.; Lage, S.; Aluwini, D.F.; Rebours, C.; Brurberg, M.B.; Nitschke, U.; Gentili, F.G. Chemical profiling of the Arctic sea lettuce *Ulva lactuca* (Chlorophyta) mass-cultivated on land under controlled conditions for food applications. *Food Chem.* **2021**, *341*, 127999.
